# Growth and applications of GeSn-related group-IV semiconductor materials

**DOI:** 10.1088/1468-6996/16/4/043502

**Published:** 2015-07-28

**Authors:** Shigeaki Zaima, Osamu Nakatsuka, Noriyuki Taoka, Masashi Kurosawa, Wakana Takeuchi, Mitsuo Sakashita

**Affiliations:** 1 EcoTopia Science Institute, Nagoya University, Furo-cho, Chikusa-ku, Nagoya 464-8603, Japan; 2 Department of Crystalline Materials Science, Graduate School of Engineering, Nagoya University, Furo-cho, Chikusa-ku, Nagoya 464-8603, Japan; 3 Innovations for High Performance Microelectronics (IHP), Im Technologiepark 25, D-15236 Frankfurt (Oder), Germany; 4 Institute for Advanced Research, Nagoya University, Furo-cho, Chikusa-ku, Nagoya 464-8601, Japan

**Keywords:** germanium tin, group-IV semiconductor, epitaxy, crystal growth, thin film, interface, energy band engineering

## Abstract

We review the technology of Ge_1−*x*_Sn_*x*_-related group-IV semiconductor materials for developing Si-based nanoelectronics. Ge_1−*x*_Sn_*x*_-related materials provide novel engineering of the crystal growth, strain structure, and energy band alignment for realising various applications not only in electronics, but also in optoelectronics. We introduce our recent achievements in the crystal growth of Ge_1−*x*_Sn_*x*_-related material thin films and the studies of the electronic properties of thin films, metals/Ge_1−*x*_Sn_*x*_, and insulators/Ge_1−*x*_Sn_*x*_ interfaces. We also review recent studies related to the crystal growth, energy band engineering, and device applications of Ge_1−*x*_Sn_*x*_-related materials, as well as the reported performances of electronic devices using Ge_1−*x*_Sn_*x*_ related materials.

## 1. Introduction

Silicon (Si) nanoelectronics have been developed for a long time, and the performance of Si ultra-large-scale integrated circuits (ULSI) has improved with the increasing integration of elementary devices with shrinking sizes, such as metal–oxide–semiconductor field-effect transistors (MOSFETs). However, one troubling recent development is that device performance has not been improved with the scaling technology of MOSFETs. Hence, it is necessary to develop *post-scaling technology*, in which novel materials should be introduced for high-mobility channels, high-dielectric insulators, metal gates for controlling the work function, and low-resistance metal/semiconductor contact [1]. Nano-scale interfaces with these new materials should also be engineered in nano-scale electronic devices. In addition, new functional devices such as optoelectronics, spintronics and power-generation devices need to be integrated into Si ULSI devices to continue the innovation of Si nanoelectronics.

Germanium tin (Ge_1−*x*_Sn_*x*_)-related group-IV semiconductor materials are attractive for various electronic and optoelectronic applications. They provide (1) strain engineering as a stressor for strained Ge, (2) energy bandgap engineering by controlling the Sn content, (3) a route for realising direct-transition semiconductors like III–V compound semiconductors with increasing Sn content, (4) improvement in interface and defect properties, and (5) a reduction of the process temperature related to the crystal growth because of the low eutectic temperature of Ge–Sn alloys.

There are various electronic device applications of high-mobility channel MOSFETs with Ge_1−*x*_Sn_*x*_ source and drain stressors, high-mobility Ge_1−*x*_Sn_*x*_ channel MOSFETs and tunnel field-effect transistors (TFETs) for high-performance and low-power consumption devices, infrared (IR) wave guides, high-efficiency IR-photodetectors, IR light-emitting diodes (LEDs), and IR lasers.

Recently, we have developed a crystal growth technique for Ge_1−*x*_Sn_*x*_ thin films and investigated their crystalline, electronic and optic properties. There have been some previous reviews of the science and engineering of Ge_1−*x*_Sn_*x*_ related materials for electronic applications [2, 3]. In this report, we review the research on Ge_1−*x*_Sn_*x*_-related technologies and our recent achievements in crystal growth, interface engineering, and the electronic and optoelectronic properties of Ge_1−*x*_Sn_*x*_ thin layers.

## 2. Energy band structure of Ge_1−*x*_Sn_*x*_-related materials

The energy band structure of Ge_1−*x*_Sn_*x*_ alloys can be controlled by adjusting the Sn content. Ge has a lower energy indirect bandgap (0.66 eV at room temperature (RT)) than Si (1.12 eV at RT). The introduction of Sn into Ge can lower the energy band gap below that of Ge. In addition, it is known that Ge_1−*x*_Sn_*x*_ becomes a direct transition semiconductor material with increasing Sn content up to about 8%. There are many reports of theoretical calculations of the energy band structure of Ge_1−*x*_Sn_*x*_ alloys [4–6].

Bulk Ge is an indirect-gap semiconductor because the conduction band edge at the L-point is at a minimum, which is 0.14 eV lower than the *Γ*-point at RT. Conversely, with increasing Sn content in Ge_1−*x*_Sn_*x*_ alloys, the conduction band edge at the *Γ*-point lowers more rapidly than that at the L-point. Then, the direct-indirect crossover of the conduction band edge occurs at an Sn content of approximately 8%, where the conduction band edge at the *Γ*-point becomes the minimum among all valleys. As a result, the minimum energy of the conduction band edge at the *Γ*-point promises a significant improvement in photoemission with the direct band transition and photo absorption with the generation of an electron-hole pair in direct emission at the *Γ*-valley. We also expect an improvement in the electron mobility because of the smaller effective mass of electrons at the *Γ*-valley than at the L-valley in Ge.

The strain in Ge and Ge_1−*x*_Sn_*x*_ also influences the energy band structure and electronic properties. The energy band structure is modified with tensile or compressive strain in Ge and Ge_1−*x*_Sn_*x*_. There are many reports of the strain and Sn content dependence of the energy band gap and band structures estimated using optical transmittance spectroscopy [6], spectroscopic ellipsometry [7], Fourier transform infrared spectroscopy (FT-IR) [8], photoluminescence [9–11] and photoreflectance spectroscopy [12]. The Sn content of the direct–indirect crossover point increases (decreases) with the magnitude of the compressive (tensile) strain [13]. One of the critical issues for the prediction of the energy band structure is the large bowing effect of the energy band edge in Ge_1−*x*_Sn_*x*_. The energy bandgap of Ge_1−*x*_Sn_*x*_ shows a significant deviation from Vegard’s law, which is a linear relationship between those of Ge and Sn. The lattice mismatch of *α*-Sn and Ge is very large, at 16%, and the local strain around Sn atoms in the Ge matrix contributes to the large bandgap bowing. There have been some studies of the bowing parameters for the theoretical prediction of energy band structures [14, 15].

We also investigated the energy bandgap of Ge_1−*x*_Sn_*x*_ with various Sn contents ranging from 0 to 23% [16]. We estimated the practical values of the energy bandgap of Ge_1−*x*_Sn_*x*_ epitaxial layers prepared on Ge(001) and InP(001) from the absorption coefficient spectra measured using FT-IR. The energy bandgap decreased from 0.66 to 0.29 eV with increasing Sn content from 5.3 to 22.7% at RT. From our FT-IR measurement, the energy bandgap bowing parameter, *b*, was determined to be *b* = −4.77 *x* + 2.47 (eV). Chibane *et al* reported the theoretical prediction of the band gap bowing parameter ranging from 2.9 to 1.8 eV for Ge_1−*x*_Sn_*x*_ with an Sn content ranging from 6.25 to 18.75% [14]. In our results, the bowing parameter was calculated to be in the range of 2.2–1.6 eV for all Sn contents. The theoretically predicted values are slightly larger, but agree well with the experimental values.

## 3. Crystal growth and crystalline properties

### 3.1. Challenges in Ge_1−*x*_Sn_*x*_ crystal growth

There are many challenges in the crystal growth of Ge_1−*x*_Sn_*x*_ layers on single-crystal Si or Ge substrate and insulators. One of the serious problems in Ge_1−*x*_Sn_*x*_ growth with an effective Sn content is Sn precipitation from Ge_1−*x*_Sn alloys. The Ge–Sn binary system is a eutectic alloy, and the thermal equilibrium solid solubility of Sn in the Ge matrix is as low as 1 at.% below 500 °C [17]. Additionally, the eutectic temperature of Ge–Sn binary alloys is as low as 231.1 °C. As a result, it is difficult to increase the Sn content in Ge_1−*x*_Sn_*x*_ because the Sn precipitation from the Ge_1−*x*_Sn_*x*_ matrix easily occurs at a low temperature during the crystal growth and post-growth processes. This difficulty contrasts to the fact that we can freely control the content of Si_1−*x*_Ge_*x*_ binary alloys, as the Si–Ge binary system has complete solid solubility.

The precipitation of Sn from Ge_1−*x*_Sn_*x*_ easily occurs during the formation of Ge_1−*x*_Sn_*x*_ layers and thermal processes for device fabrications [18, 19]. It is necessary to control the Sn precipitation with non-thermal equilibrium processes such as low-temperature processes, strain engineering of thin films and the introduction of third elements to compensate for the local strain around an Sn atom. Sn segregation during the growth and post-processing is one of the problems associated with the formation of homogeneous Ge_1−*x*_Sn_*x*_ layers. We found that Sn segregation occurs during the epitaxial growth of Ge_1−*x*−*y*_Si_*x*_Sn_*y*_ ternary alloys [20]. Sn segregation also occurs during the oxidation of Ge_1−*x*_Sn_*x*_ layers. The diffusion of Sn is enhanced with the oxidation of Ge_1−*x*_Sn_*x*_, and an increase in the Sn content near the surface with Sn segregation is observed after the oxidation of Ge_1−*x*_Sn_*x*_ layers [21].

Therefore, we need to consider how to increase the Sn content over the critical value of Sn precipitation by engineering the crystalline properties such as the thermodynamics of the bulk, interface, and strain energy, and the kinetics of the diffusion, segregation, and nucleation of Sn atoms in Ge_1−*x*_Sn_*x*_. Non-equilibrium processes are also effective for suppressing the Sn precipitation. We have developed the low-temperature growth of Ge_1−*x*_Sn_*x*_, which is demonstrated below.

### 3.2. Molecular beam epitaxy (MBE) growth of Ge_1−x_Sn_x_ epitaxial layers

MBE is one of the major preparation methods for high-quality Ge_1−*x*_Sn_*x*_ layers on Si, Ge, and other substrates. In the early stages, there were some reports of the MBE growth of Ge_1−*x*_Sn_*x*_ epitaxial layers on Si or Ge substrates [22–26]. Ge and Sn were usually deposited with Knudsen cells in an ultra-high vacuum (UHV) chamber. The calculated equilibrium vapor pressure of Sn is high, at 1.3 × 10^−10^ and 6 × 10^−5^ Pa at 500 and 800 °C, respectively [27], while the melting point of Sn is very low, at 231.9 °C. Hence, a crucible temperature as high as 800 °C is often required to obtain a sufficiently high deposition rate.

The substrate temperature for MBE growth is key for obtaining a high-quality Ge_1−*x*_Sn_*x*_ layer without Sn precipitation. The commonly used growth temperature of 400 °C for Ge epitaxy is slightly high for Ge_1−*x*_Sn_*x*_ growth, and Sn precipitation is often observed at a high growth temperature. The growth temperature should be reduced to as low as the eutectic temperature, 231.1 °C, to avoid Sn precipitation. Low-temperature growth can effectively suppress Sn precipitation. We have demonstrated that the low-temperature MBE growth of Ge_1−*x*_Sn_*x*_ epitaxial layers with high Sn contents of 9 and 12% can be achieved on Si(001) and Ge(001) substrates, respectively [28, 29]. The non-thermal equilibrium growth of Ge_1−*x*_Sn_*x*_ heteroepitaxy realizes the introduction of Sn atoms with contents larger than the thermal equilibrium solid-solubility into substitutional sites in the Ge matrix.

**Figure 1.**
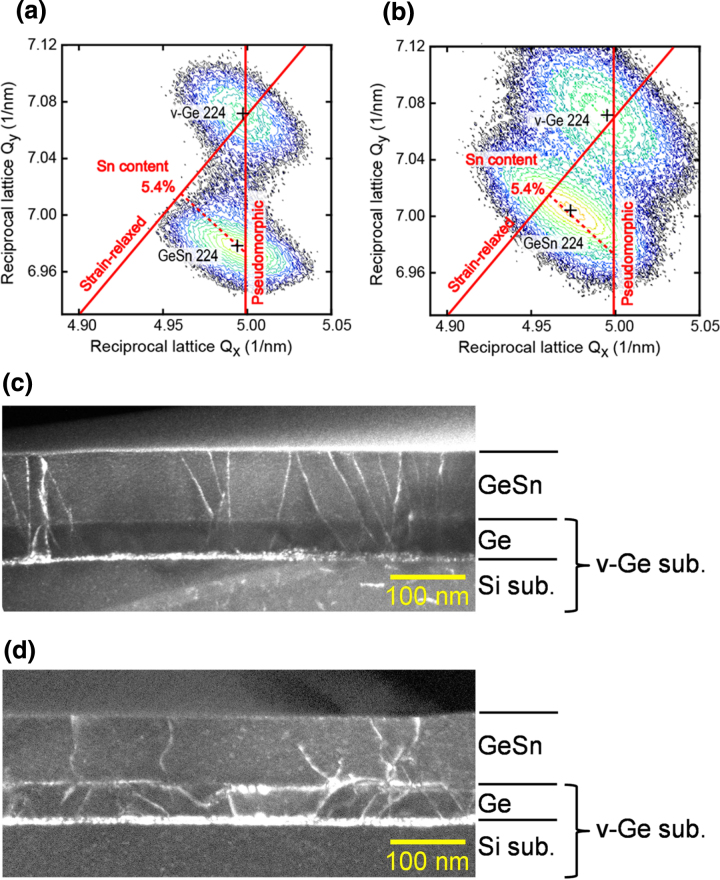
XRD-2DRSM around the Ge 224 reciprocal lattice point for Ge_1−*x*_Sn_*x*_ epitaxial layers grown on the virtual Ge substrate for samples after (a) the growth and (b) the PDA treatment. Cross sectional TEM images of Ge_1−*x*_Sn_*x*_ epitaxial layers grown on the virtual Ge substrate for samples after (c) the growth and (d) the PDA treatment.

Figures 1(a) and (b) show the typical x-ray diffraction two-dimensional reciprocal space maps (XRD-2DRSM) around the Ge 224 reciprocal lattice point for Ge_1−*x*_Sn_*x*_ layers on a virtual Ge (v-Ge) substrate for samples after growth and post-deposition annealing (PDA). v-Ge substrate means a substrate that consists of a strain relaxed Ge layer epitaxially grown on an Si substrate. In these samples, Si(001) wafers were used as substrates. The vertical straight line through the v-Ge 224 reciprocal lattice point in 2DRSM indicates the trajectory of the 224 reciprocal lattice point of an epitaxial layer *pseudomorphically* grown on a v-Ge substrate. The diagonal line through the v-Ge 224 point also indicates the trajectory of the 224 reciprocal lattice point of a cubic structure, meaning a *strain-relaxed* epitaxial layer. From the peak position of the Bragg reflection related to the Ge_1−*x*_Sn_*x*_ layer in XRD-2DRSM, we can estimate the content of substitutional Sn atoms and the degree of strain relaxation of the Ge_1−*x*_Sn_*x*_ epitaxial layer. In the as-grown sample, the Ge_1−*x*_Sn_*x*_ layer is pseudomorphically grown, with lattice matching with the v-Ge substrate. After the PDA treatment at 500 °C for 10 min, we can see the movement of the peak position, meaning the strain relaxation of the Ge_1−*x*_Sn_*x*_ layer with introduced misfit dislocations. We can also confirm the absence of Sn precipitation from the Ge_1−*x*_Sn_*x*_ layer by estimating the Sn content from the diffraction peak position.

Figures 1(c) and (d) show cross-sectional transmission microscopy (TEM) images of Ge_1−*x*_Sn_*x*_ layers on v-Ge before and after PDA treatment, respectively, for the samples shown in figures 1(a) and (b). In the pseudomorphic Ge_1−*x*_Sn_*x*_ layer grown on v-Ge substrate, we can see threading dislocations propagating from those in the strain relaxed Ge layer, although we can see no contrast at the Ge_1−*x*_Sn_*x*_/v-Ge interface. After PDA treatment, a clear contrast appears at the interface, which indicates the propagation of misfit dislocations, causing the strain relaxation.

**Figure 2.**
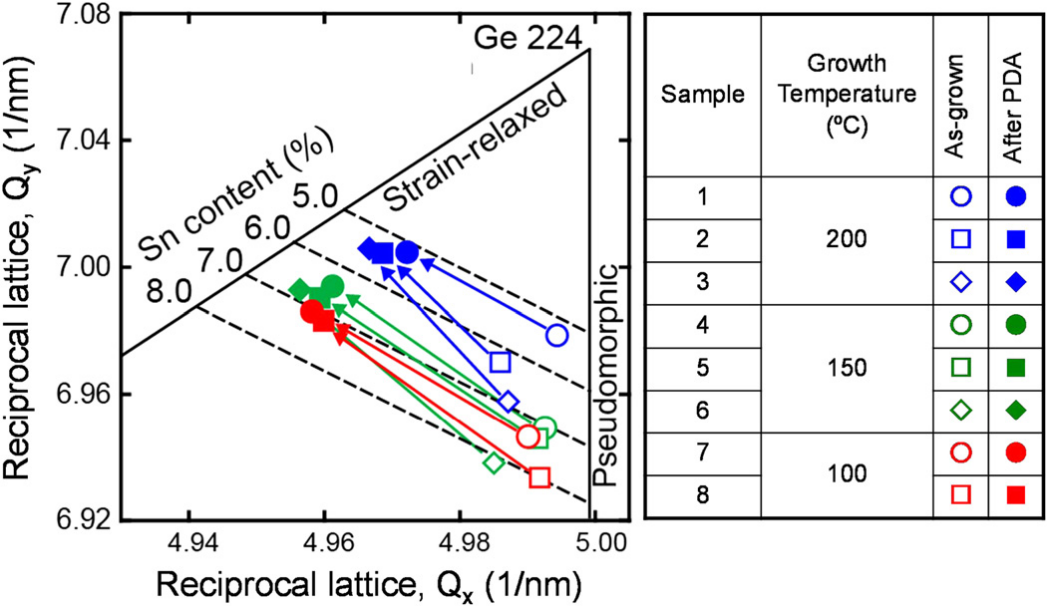
Summary of XRD-2DRSM results for Ge_1−*x*_Sn_*x*_ layers with various Sn contents after the growth at 100, 150, and 200 °C. Reprinted from [28], Copyright 2009, with permission from Elsevier.

Figure 2 shows a summary of XRD-2DRSM results for Ge_1−*x*_Sn_*x*_ layers with various Sn contents after growth at 100, 150, and 200 °C [28]. The Sn contents of all samples grown at 200 °C after PDA were not higher than 5.5% because of Sn precipitation. In contrast, in the samples grown at 150 and 100 °C, high Sn contents of 6.8 and 7.1%, respectively, were achieved, even after PDA at 500 °C. This result indicates that Sn precipitation can be suppressed by lowering the growth temperature. The Sn precipitation is thought to be suppressed because of the effects of point defects of vacancies introduced into the Ge_1−*x*_Sn_*x*_ epitaxial layer during the low temperature MBE growth. One reason is the reduction of the strain in the Ge_1−*x*_Sn_*x*_ layer with increasing lateral propagation of misfit dislocations by the introduction of point defects in the Ge_1−*x*_Sn_*x*_ layers with lower growth temperature. Another reason is the reduction of the local strain around an Sn atom when binding a vacancy defect introduced by lowering the growth temperature. The substitutional Sn atom will be energetically stabilised by the formation of an Sn–vacancy (Sn–V) pair in the Ge matrix [30].

In addition, the strain in Ge_1−*x*_Sn_*x*_ layers strongly influences the Sn precipitation. We previously found that there is a critical misfit value between a biaxially compressive strained Ge_1−*x*_Sn_*x*_ and buffer layers for Sn precipitation [31, 32]. The Sn precipitation can be suppressed by Ge_1−*x*_Sn_*x*_ epitaxy with a misfit value below 0.37% in the case of the growth at 200 °C; above the misfit value, the Sn precipitation occurs and as a result the strain in the epitaxial layer is reduced. We demonstrated the step-graded growth of three stacked Ge_1−*x*_Sn_*x*_ epitaxial layers, in which the Sn content increases from 1.0 to 6.7% for gradually increasing in the lattice constant of Ge_1−*x*_Sn_*x*_ layers with keeping a misfit value between upper and lower layers as low as possible [31]. The step-graded growth of Ge_1−*x*_Sn_*x*_ layers effectively suppresses the Sn precipitation, which can achieve a strain-relaxed Ge_1−*x*_Sn_*x*_ layer with an Sn content as high as 6.3% on v-Ge substrate.

Considering that low-temperature growth and the reduction of the misfit value between the Ge_1−*x*_Sn_*x*_ layer and substrate are key to suppressing the Sn precipitation, misfit control by increasing the lattice constant of the substrate for Ge_1−*x*_Sn_*x*_ epitaxy with very high Sn content may be a potential solution. We can achieve the epitaxial growth of Ge_1−*x*_Sn_*x*_ with an ultra-high Sn content of 27% with low-temperature MBE growth of Ge_1−*x*_Sn_*x*_ on InP [33]. InP has a lattice constant of 0.58686 nm, which is larger than that of Ge and matches that of a Ge_1−*x*_Sn_*x*_ layer with an Sn content as high as 25.4%. We demonstrated the low-temperature growth of a Ge_1−*x*_Sn_*x*_ layer at 50 °C, and achieved a Ge_1−*x*_Sn_*x*_ epitaxial layer with an Sn content of 27%, while that was three-dimensional growth with many stacking faults.

**Figure 3.**
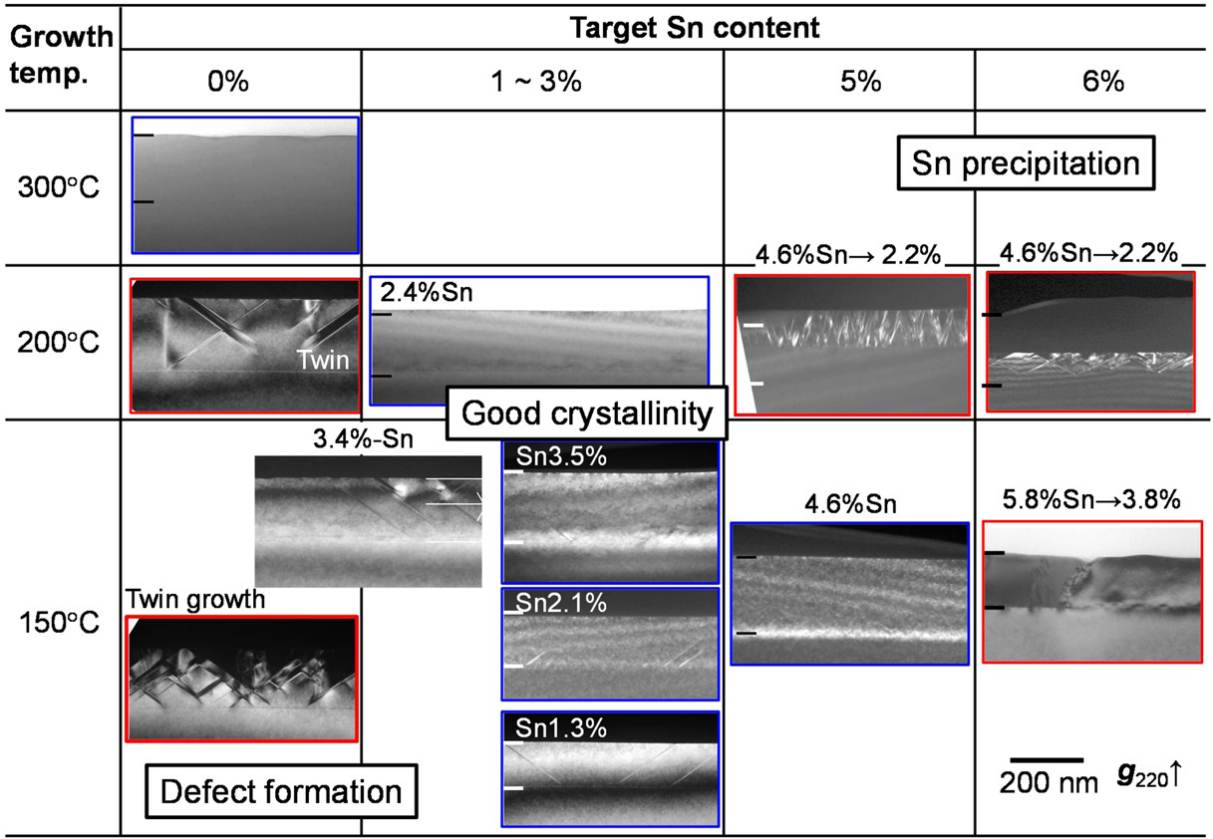
The summary of cross-sectional TEM images of Ge_1−*x*_Sn_*x*_/Ge(110) samples grown at 150, 200, and 300 °C with Sn contents ranging from 0 to 6%.

The Ge_1−*x*_Sn_*x*_ epitaxy on substrates other than those with (001) orientation is attractive for applications involving high-mobility transistors and optoelectronic devices. There is an issue with the epitaxial growth of Si_1−*x*_Ge_*x*_ or Ge on (110) substrates because stacking faults and twin defects are often introduced, especially with low-temperature growth [34, 35]. However, we found that the introduction of Sn into Ge epitaxy effectively improved the crystalline quality of the epitaxial layer on Ge(110) or Ge(111), even at a low temperature of 150 °C [36–40]. Figure 3 shows a summary of cross-sectional TEM images of Ge_1−*x*_Sn_*x*_ epitaxial layers grown on Ge(110) substrates at various temperatures with various Sn contents. Homoepitaxy of Ge layers on Ge(110) at a growth temperature below 200 °C causes the introduction of many stacking faults and twin defect formation. In contrast, the twin defects and stacking faults in a Ge epitaxial layer are significantly suppressed with the introduction of Sn with a content of 5%, even at a growth temperature as low as 150 °C. [36, 37]. In addition, the length of stacking faults in the Ge_1−*x*_Sn_*x*_ epitaxial layer decreases with increasing Sn content. It is possible that the increase in the misfit strain between Ge_1−*x*_Sn_*x*_ and Ge(110) contributed to preventing the growth of stacking faults, according to the elastic theory for epitaxial layer [41].

Interestingly, Ge_1−*x*_Sn_*x*_ epitaxy on Si(110) substrate is also improved compared with Ge heteroepitaxy on Si(110). We found that twin defect growth is significantly avoided with the introduction of Sn with a content as low as 2%, even with low-temperature growth at 200 °C [42, 43]. The surface roughness is improved by suppressing twin growth and changing the growth mode. The density of threading dislocations also effectively decreases with PDA because of the enhancement of the propagation of misfit dislocations. The modification of the epitaxial growth with the introduction of Sn promises to extend the applications of Ge and Ge_1−*x*_Sn_*x*_ for Si-based nanoelectronics.

### 3.3. Chemical vapor deposition (CVD) growth of Ge_1−*x*_Sn_*x*_ epitaxial layers

CVD for the formation of Ge_1−*x*_Sn_*x*_ epitaxial layers has also been actively developed. The CVD method is one of the most important technologies because it promises uniform and homogeneous formation of Ge_1−*x*_Sn_*x*_ layers on large substrates for the mass production of Ge_1−*x*_Sn_*x*_ applications integrated into Si ULSI devices. There have been some previous reports on the CVD growth of Ge_1−*x*_Sn_*x*_ epitaxial layers on Si and Ge substrates.

The Ge precursor Ge_2_H_6_ is usually used in the CVD growth for Ge_1−*x*_Sn_*x*_ epitaxy, similar to a conventional source gas in Ge CVD. One of the important factors for the CVD growth of Ge_1−*x*_Sn_*x*_ layers is selecting a suitable Sn precursor. Kouvetakis’s group proposed using SnD_4_ as a precursor because SnD_4_ is more stable than SnH_4_, which has autoproteolysis characteristics. They reported the epitaxial growth of unstrained Ge_1−*x*_Sn_*x*_ layers with an Sn content from 2 to 15% on Si(001) substrates using the low-pressure CVD method at 250–350 °C with a combination of the precursors SnD_4_ and Ge_2_H_6_ [44–46]. They also reported CVD growth with the combination of Ge_3_H_8_ and SnD_4_ precursors for lowering the growth temperature of Ge_1−*x*_Sn_*x*_ layers [11]. Vincent *et al* reported the epitaxial growth of Ge_1−*x*_Sn_*x*_ layers on a Ge(001) substrate using an atmospheric pressure-CVD method. They used Ge_2_H_6_ and SnCl_4_ as Ge and Sn precursors, respectively. The advantage of SnCl_4_ is the total absence of instability issues compared with SnD_4_ and the fact that it is a commercially available product. The epitaxial growth of a Ge_1−*x*_Sn_*x*_ layer with an Sn content of 8% was achieved at a low temperature of 320 °C [47].

Metal organic CVD (MOCVD) is an attractive growth technique for many applications because of its low cost and safety. Precursor sources for MOCVD are comparably safe from the view point of such dangerous characteristics as explodability, hypergolicity, and toxicity compared with other CVD sources such as GeH_6_, SnD_4_, and SnCl_4_. Ogura’s group proposed metal-organic sources of tertiary-butyl-germane (TBGe), t-C_4_H_9_GeH_3_, and tetra-ethyl-tin (TESn), (C_2_H_5_)_4_Sn, as precursors for Ge and Sn, respectively, for MOCVD [48, 49]. They examined the growth of Ge_1−*x*_Sn_*x*_ layers on a Si substrate using the MOCVD method at 350–380 °C. Recently, we demonstrated the epitaxial growth of Ge_1−*x*_Sn_*x*_ layers on a Ge(001) substrate using the MOCVD method [50, 51]. In our study, we used the precursors of TBGe and tri-butyl-vinyl-tin, t-C_2_H_9_SnCH_2_. A Ge_1−*x*_Sn_*x*_ epitaxial layer with an Sn content of 5.1% was achieved using MOCVD at 300 °C. The surface morphology and crystalline structures of Ge_1−*x*_Sn_*x*_ epitaxial layers prepared using the MOCVD method are superior compared to those of Ge_1−*x*_Sn_*x*_ layers grown with the MBE method [51]. In the Ge_1−*x*_Sn_*x*_ layer prepared with low-temperature MBE growth, we can often see inhomogeneous contrast in the cross-sectional TEM observation, which indicates the fluctuation of the lattice constant of Ge_1−*x*_Sn_*x*_ layer. Conversely, the TEM image of the Ge_1−*x*_Sn_*x*_ layer grown by MOCVD shows uniform contrast. This should be attributed to the surfactant effect of hydrogen during the Ge_1−*x*_Sn_*x*_ growth [52].

### 3.4. Solid-phase crystallization (SPC) technology of Ge_1−*x*_Sn_*x*_

Low-temperature formation of group-IV materials such as Si, Ge and their mixed films on insulating surfaces has been expected to open up a higher degree of freedom for the fabrication of devices such as high-speed thin-film transistors (TFTs), high-efficiency thin-film solar cells, and 3D integrated circuits (ICs). To prevent the softening of glass substrates and interference with the fabrication process for 3D circuits, the process temperature for polycrystallization of these materials should be lower than approximately 450 °C. In this respect, the abovementioned Ge_1−*x*_Sn_*x*_ is an interesting material because the process temperature can be significantly suppressed compared with that of a conventional Si process, which is attributed to the very low melting point of Sn (231.9 °C).

Because tin can be a semiconductor (*α*-Sn) and a metal (*β*-Sn), it can be used as a catalyst for the polycrystallization of amorphous semiconducting films of Ge. If almost all of the Sn atoms could be incorporated substitutionally into the Ge lattice after the polycrystallization, the grown Ge_1−*x*_Sn_*x*_ films would show a high mobility, which is a great advantage compared with conventional metal-induced crystallizations (MIC) using catalysts such as Ni, Al, and Cu. However, thin film crystallization applying the low melting temperature and solubility of Sn had not been conducted until recently. Under such a background, we investigated the growth of Ge_1−*x*_Sn_*x*_-related polycrystalline materials on insulators 3 years previously. In this section, we will present the feasibility of poly-Ge_1−*x*_Sn_*x*_ device fabrication on ULSI circuits and/or plastic substrates using a low-temperature process through our recent research results.

**Figure 4.**
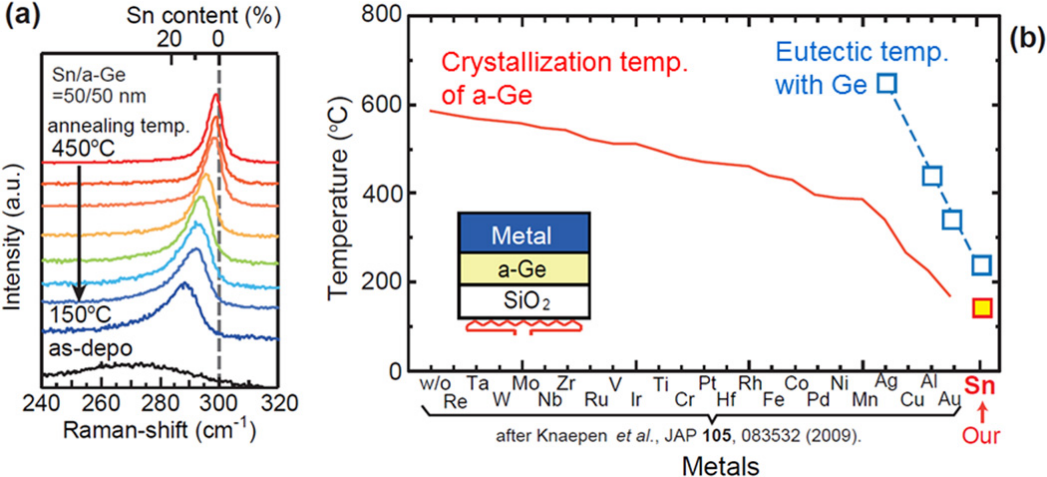
(a) Raman spectra obtained from Sn/a-Ge stacked structures after annealing. (b) Temperature of metal-induced crystallization using various metals.

To confirm the effect of Sn on the polycrystallization temperature of amorphous Ge (a-Ge), we first conducted verification experiments for Sn-induced crystallization of a-Ge using stacked structures of Sn/a-Ge films with thicknesses of 50/50 nm [53]. The initial sample structure and typical Raman spectra after annealing for 5 h at various temperatures are shown in figure 4(a). The sharp Raman peaks resulting from crystallization of the films are clearly observed, even at 150 °C. In addition, the peak positions shifted to lower wavenumbers with decreasing annealing temperatures from 450 to 150 °C, indicating an increase in substitutional Sn content in the poly-Ge_1−*x*_Sn_*x*_ from ∼2% to ∼13%. The temperature of Sn-induced crystallization of Ge is summarized in figure 4(b), together with various MIC results reported by Knaepen *et al* [54], where the MIC temperatures are sorted in descending order. Interestingly, lower crystallization temperatures were obtained for the eutectic metals Al, Au, and Sn.

In recent years, eutectic-metal induced crystallization has become a hot topic because it has the possibility to obtain not only low-temperature crystallization but also orientation control of Si_1−*x*_Ge_*x*_ films on insulators. Research groups at Kyushu University and Tsukuba University have individually studied the eutectic-metal induced crystallization of Si [55], Ge [56, 57], and Si_1−*x*_Ge_*x*_ [58, 59], aiming for orientation control. From a technological point of views, Park *et al* [60] and Toko *et al* [61] individually demonstrated the sub-200 °C formation of highly (111)-oriented poly-Ge films on flexible plastic substrates. Surprisingly, the (111)Ge films grown by Au-induced crystallization have a very high hole mobility, 160 cm^2^ V^−1^ s^−1^ at 300 K [62]. This is good news for TFTs. In addition, very recently, Kurosawa *et al* [63] proposed a comprehensive oriented-growth model that well explains many groups’ results for eutectic-metal induced crystallization from the scientific point of views. We look forward to further progress in device fabrication, aiming for high-performance TFTs and thin-film solar cells.

**Figure 5.**
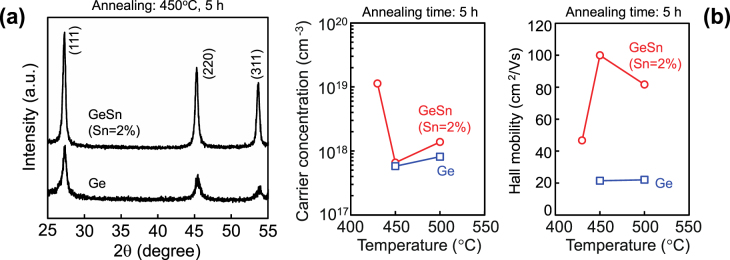
(a) XRD profiles of Ge and Ge_1−*x*_Sn_*x*_ films annealed at 450 °C for 5 h. (b) Carrier concentrations and Hall mobilities for Ge layer and Ge_1−*x*_Sn_*x*_ films after annealing at various temperatures.

We recently reported that holes were generated in epitaxial Ge_1−*x*_Sn_*x*_ films (*x* = 0 – 0.058) grown on Si on insulator (SOI) using MBE. The hole concentration increased with the Sn content and reached high values (3 × 10^18^ cm^−3^) at *x* = 0.058, with a mobility of ∼100 cm^2^ V^−1^ s^−1^ at 300 K [64]. Consequently, we selected the incorporated Sn content to be 2% for SPC [65]. The typical SPC results are summarized in figure 5, where 300 nm thick Ge or Ge_1−*x*_Sn_*x*_ films with an initial Sn content of 2% were used. Sharper diffraction peaks for the Ge_1−*x*_Sn_*x*_ film were clearly observed compared with those for the Ge film. The full width at half maximum for the Ge_1−*x*_Sn_*x*_ film (∼0.4°) is smaller than that for the Ge film (∼0.6°), suggesting that the Ge_1−*x*_Sn_*x*_ film has larger grains or higher crystallinity compared with the Ge film.

Correspondingly, the smallest carrier concentration (7 × 10^17^ cm^−3^) and highest Hall mobility (∼100 cm^2^ V^−1^ s^−1^) were obtained for Ge_1−*x*_Sn_*x*_ films annealed at 450 °C for 5 h. It should be noted that the mobility of the Ge_1−*x*_Sn_*x*_ film annealed at 450 °C for 5 h is five times larger than that for the Ge film. In addition, the obtained mobility in this experiment is almost the same mobility (100–140 cm^2^ V^−1^ s^−1^) as that for poly-Ge channel TFTs reported by Sadoh *et al* where the maximum process temperature was 500 °C in the SPC of a-Ge films [66, 67]. From these results, we can say that the 2% Sn incorporation into Ge can effectively improve the mobility, especially when low-temperature annealing is used; however, it is desirable to increase the hole mobility further because the mobility is still smaller than that of single-crystalline Si. This can be achieved by obtaining larger crystallite grain sizes, in the order of a micrometre (normal device size).

**Figure 6.**
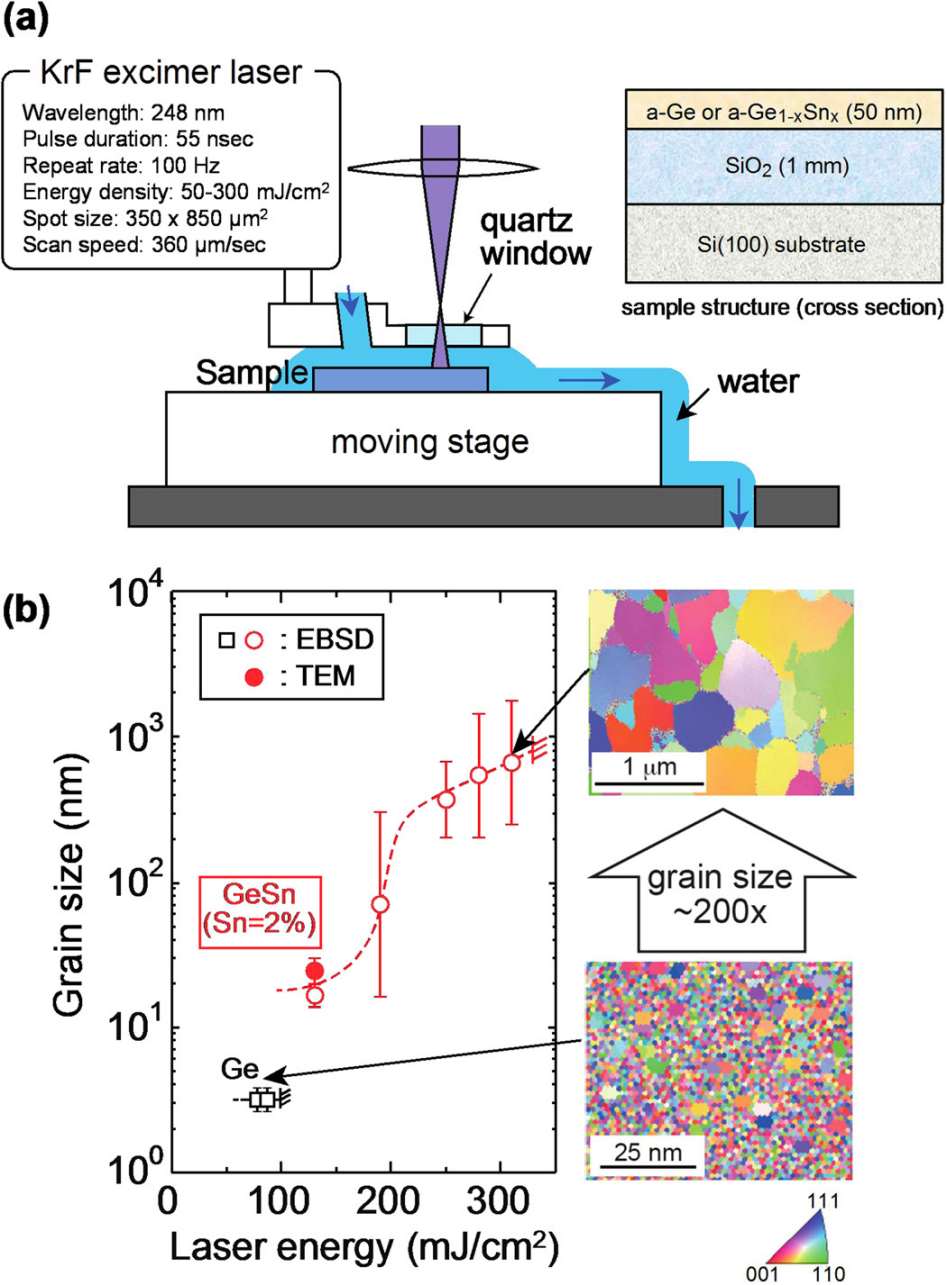
(a) Schematic illustrations of the initial sample structure and the pulsed laser annealing system. (b) Grain sizes of crystallized layers for various Ge_1−*x*_Sn_*x*_ samples as a function of laser energy. The inset in (b) shows EBSD maps of Ge films and Ge_1−*x*_Sn_*x*_ films with an Sn content of 2% after PLA in water at 85 and 310 mJ cm^−2^, respectively. Reprinted with permission from [68]. Copyright 2014, American Institute of Physics.

Pulsed laser annealing (PLA) in water is very useful for this purpose [68]. The sample structure and the experimental setup for the PLA process in water are shown in figure 6(a), respectively. a-Ge_1−*x*_Sn_*x*_ films (initial Sn contents: 0 and 2%) with a thickness of 50 nm were crystallized using a 55 ns KrF excimer laser (wavelength: 248 nm) and were irradiated in water at RT, showing that it is possible to provide local and rapid heating to crystallize the amorphous films without thermally damaging the underlying ICs and substrates. The grain size distributions after PLA in water were evaluated by electron backscatter diffraction (EBSD) as a function of the laser energy for various samples, as shown in figure 6(b). The surface roughness and ablation of the films can be suppressed by combining underwater irradiation and incorporating 2% Sn into the a-Ge films, which allows for the use of a higher energy laser. As a result, large-grain (∼800 nm) poly-Ge_1−*x*_Sn_*x*_ films were realized on SiO_2_ substrates without any Sn precipitation, as shown in the inset of figure 6(b). The resulting maximum grain size was at least seven times larger than that with conventional PLA of a-Ge in a vacuum (∼126 nm) [69].

Using PLA methods, very recently, we have successfully fabricated poly-Ge_1−*x*_Sn_*x*_ junctionless (JL) tri-gate p-type field-effect transistors (FETs) [70]. Major drawbacks in Ge_1−*x*_Sn_*x*_ channels, such as junction leakage current resulting from bandgap narrowing, have been overcome by JL FETs with tri-gates. The recorded cut-off characteristics of poly-Ge_0.97_Sn_0.03_ tri-gate p-FETs with *I*_on_/*I*_off_ > 10^5^ at *V*_d_ = −50 mV and SS = 125 mV/decade have been successfully realized by thinning the fin width down to approximately 20 nm, with results that are comparable to or better than their counterparts consisting of single-crystalline Ge_1−*x*_Sn_*x*_ (*x* = 0 – 0.07) p-FETs [71, 72]. The details of this work will be published in the near future. Furthermore, recent excellent studies of high-performance poly-Ge p- and n-FETs, and poly-Ge CMOS operation, have been conducted by Usuda *et al* [73] and Kamata *et al* [74], respectively. These studies are quite informative for realising sequential integration of poly-Ge FETs in a 3D-IC. This is the first step to realize Ge_1−*x*_Sn_*x*_-related 3D-ICs, but we believe that poly-Ge_1−*x*_Sn_*x*_ FETs will become an option to create 3D-ICs in next-generation ULSI.

### 3.5. Ternary alloys of Ge_1−*x*_Sn_*x*_-related materials

**Figure 7.**
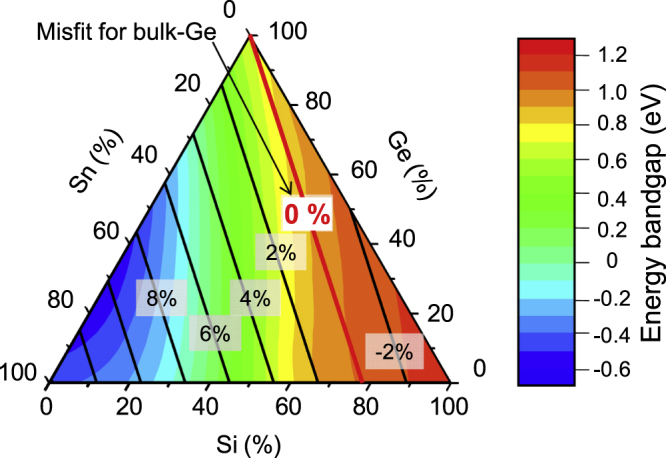
Ternary diagram of the energy bandgap of Ge_1−*x*−*y*_Si_*x*_Sn_*y*_ ternary alloy with various contents of Ge (0% ∼ 100%), Si (0% ∼ 100%), and *α*-Sn (0% ∼ 100%). The energy bandgap was estimated by considering the conduction band edges at L- and *Γ*-valleys according a previous report of [77]. The solid lines in the diagram are the trajectories for the lattice mismatching cases for bulk-Ge.

Ternary alloy group-IV semiconductors are also attractive materials for electronic and optoelectronic applications. Ge_1−*x*−*y*_Si_*x*_Sn_*y*_ ternary alloys are expected to use the buffer material of epitaxial layers of group-III–V compound semiconductors for multi-junction solar cell structures [75]. Ge_1−*x*−*y*_Si_*x*_Sn_*y*_ also holds promise for energy band engineered materials such as TFETs and quantum well (QW) lasers because the energy bandgap can be controlled independently of the lattice constant by changing the relative contents of the three elements of Ge_1−*x*−*y*_Si_*x*_Sn_*y*_. There have been some theoretical predictions of the energy band structure of Ge_1−*x*−*y*_Si_*x*_Sn_*y*_ [76–79]. Figure 7 shows a ternary diagram of the energy bandgap of Ge_1−*x*−*y*_Si_*x*_Sn_*y*_ with varying contents of Ge (0–100%), Si (0–100%), and *α*-Sn (0–100%). Here, we consider some parameters of *α*-Sn. The energy bandgap was estimated by considering the conduction band edges at L- and *Γ*-valleys at RT according to the theoretical calculation previously reported by Moontragoon *et al* in which they provide bowing parameters for the bandgap calculation by using empirical pseudopotential theory [77]. The solid lines in the diagram show the trajectories for some lattice mismatching cases for bulk Ge, which were calculated assuming Vegard’s law. The energy bandgap of Ge_1−*x*−*y*_Si_*x*_Sn_*y*_ ternary alloys can be controlled by maintaining a constant lattice mismatch value.

The crystal growth of Ge_1−*x*−*y*_Si_*x*_Sn_*y*_ ternary alloy thin films with MBE [80, 81] and CVD [46, 75] methods has been reported. Recently, we reported the epitaxial growth of Ge_1−*x*−*y*_Si_*x*_Sn_*y*_ layers whose lattice matches that of a Ge(001) substrate using the MBE method [81, 82]. The crystalline structure of lattice-matching Ge_1−*x*−*y*_Si_*x*_Sn_*y*_ epitaxial layers on Ge is often superior to that of strained Ge_1−*x*_Sn_*x*_ layers on Ge substrates because the effect of strain is minimized. Lattice tilting because of the strain relaxation related to the lattice mismatching is significantly suppressed, even with Sn contents higher than 15%, while large tilting is observed in the epitaxial layer of Ge_1−*x*_Sn_*x*_ binary alloys on Ge substrates. In addition, the thermal stability of substitutional Sn atoms in the Ge_1−*x*−*y*_Si_*x*_Sn_*y*_ layers is significantly high compared with that in Ge_1−*x*_Sn_*x*_ epitaxial layers. Sn precipitation rarely occurs, even with a high Sn content of 7% and a high annealing temperature of 600 °C [83]. The high stability of substitutional Sn atoms in Ge_1−*x*−*y*_Si_*x*_Sn_*y*_ epitaxial layers can be attributed to the compensation of the local strain around large Sn atoms in the Ge matrix with Si atoms whose lattice constant is smaller than that of Ge.

Recently, we found that the sign of strain, meaning compressive or tensile, strongly affects the epitaxial growth of Ge_1−*x*−*y*_Si_*x*_Sn_*y*_ alloy layers [82]. The tensile strain in Ge_1−*x*−*y*_Si_*x*_Sn_*y*_ epitaxial layers on Ge(001) induces a non-uniform crystallinity of (220) lattice planes and surface roughening, even though the strain magnitude is not as high as 0.2%. In contrast, an unstrained or compressively strained Ge_1−*x*−*y*_Si_*x*_Sn_*y*_ epitaxial layer exhibits a flat and uniform surface and high crystallinity. This fact means that we have to take account of not only the magnitude of the strain but also the sign of the strain for low-temperature epitaxy of high-quality Ge_1−*x*−*y*_Si_*x*_Sn_*y*_ layers.

Ge_1−*x*−*y*_Si_*x*_Sn_*y*_ ternary alloys hold promise for contributing to the realization of type-I energy band structures combined with Ge or Ge_1−*x*_Sn_*x*_. A type-I structure is a very important energy band structure for carrier confinement in electronic and optoelectronic applications such as high-electron mobility transistors, LEDs, QW lasers, and photovoltaics. Type-I structures are usually realized with III–V compound semiconductors such as GaAs/AlAs, or strained systems of group-IV semiconductors, such as strained Si_1−*x*_Ge_*x*_/Si. Some theoretical calculations have discussed the realization of type-I structures with a Ge/Ge_1−*x*−*y*_Si_*x*_Sn_*y*_ system [76, 84, 85]. This means there is a possibility of realising the type-I energy band structure with only group-IV semiconductor materials maintaining unstrained or very-small-strained structures, which extends the application potential of Ge, Ge_1−*x*_Sn_*x*_, and Ge_1−*x*−*y*_Si_*x*_Sn_*y*_ materials.

Recently, we also examined other group-IV ternary alloys of Sn-related materials, Ge_1−*x*−*y*_Sn_*x*_C_*y*_ and Si_1−*x*−*y*_Sn_*x*_C_*y*_ [86–88]. The challenge in the crystal growth of these materials is how to increase the content of the substitutional C atoms. The ternary alloying for these combinations provides a positive effect, which is the compensation of the local strain between Sn and C in Ge or Si matrices. The content of substitutional C can be successfully enhanced in ternary alloys of Ge_1−*x*−*y*_Sn_*x*_C_*y*_ and Si_1−*x*−*y*_Sn_*x*_C_*y*_ compared with the binary alloys Ge_1−*x*_C_*x*_ and Si_1−*x*_C_*x*_. This result is supported by theoretical calculations of the energetic stability of Sn–C pairs in Ge and Si matrices [89].

## 4. Heterostructures, interfaces, and defect properties

### 4.1. Passivation techniques for Ge_1−x_Sn_x_ MOS interfaces

As previously mentioned, Sn has a quite small solid solubility limit in Ge [17, 90] and Si [91] and a small surface energy compared with Ge [92, 93]. Therefore, Sn atoms often precipitate in a GeSn(Si) layer and segregate on a GeSn(Si) surface during deposition and annealing after GeSn(Si) layer formation (referred to as PDA) [20, 21, 94]. Furthermore, high deposition temperatures and PDA at high temperatures induce Sn desorption from the surface [20, 21, 95]. These results mean that MOS technology for an Sn-based alloy is a challenging technology because a technique to precisely control the physical behaviors of Sn atoms during layer formation and PDA must be established. Recently, to overcome these issues, control of the MOS interface structures has been attempted by capping layer techniques, surface oxidation, and CVD. A Ge capping layer formed between an insulator film and a Ge_1−*x*_Sn_*x*_ surface is effective for reducing gate leakage current and improving MOS interface properties [96]. The reduction and improvement are attributed to avoiding Sn segregation and diffusion into the insulator film by introducing the capping layer. Additionally, an Si capping layer leads to high performance of Ge_1−*x*_Sn_*x*_ p-channel MOSFETs [97]. These results are discussed in a later section 5.1. This could be a result of the buried channel effect induced by the large energy offset at the valence band between Si and Ge_1−*x*_Sn_*x*_ as well as Ge p-channel MOSFETs with an Si passivation layer [98, 99]. These capping layer techniques make it possible to reduce the impacts of the Sn segregation. In contrast, precise control of defect introduction because of the lattice mismatch and inter-diffusion at the Si(or Ge)/Ge_1−*x*_Sn_*x*_ interface and suppression of equivalent oxide thickness are required [99, 100].

### 4.2. Oxide/Ge_1−x_Sn_x_ interface properties

Another approach is formation of an insulator/Ge_1−*x*_Sn_*x*_ interface without a semiconductor layer controlling the Sn migration. Because the heat of formation of Sn-oxide (−578 kJ mol^−1^) is almost identical to that of Ge-oxide (−580 kJ mol^−1^) [101], thermal oxidation of a Ge_1−*x*_Sn_*x*_ layer would not induce significant preferential oxidation of Ge or Sn if substitutional Sn atoms in the Ge_1−*x*_Sn_*x*_ layer are stable during the thermal oxidation. However, the robustness of the substitutional Sn atoms during thermal oxidation of a Ge_1−*x*_Sn_*x*_ surface has not been investigated yet. Therefore, we investigated the physical behaviors of Sn atoms during thermal oxidation and the impact of thermal oxidation on the electrical interface properties [21, 95].

**Figure 8.**
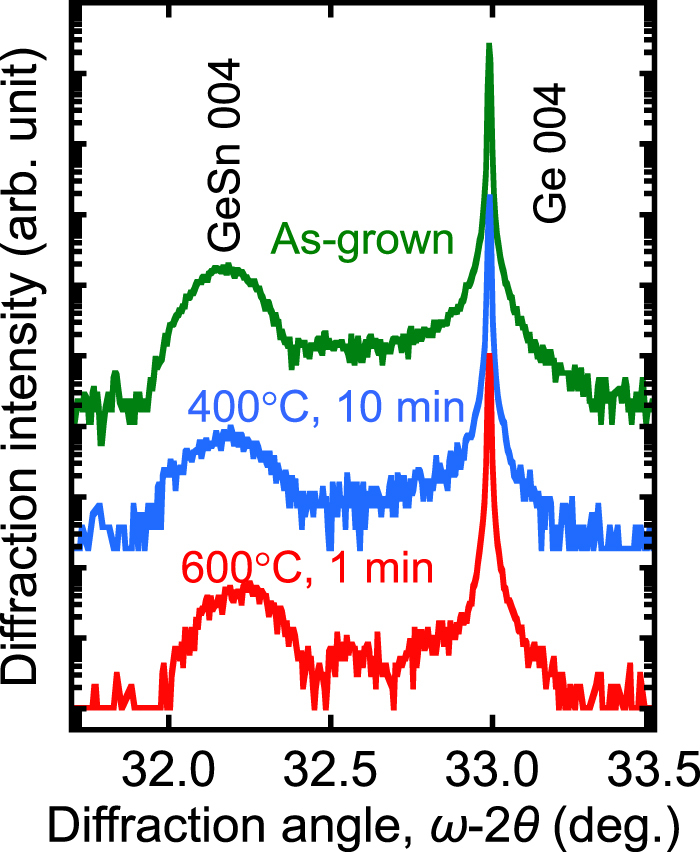
XRD *ω*-2*θ* profiles measured near the 004 diffraction plane for the as-grown Ge_1−*x*_Sn_*x*_ layer and the Ge_1−*x*_Sn_*x*_ layers oxidized at 400 and 600 °C. Here, *C*_Sn-XRD_ is 8.7.

**Figure 9.**
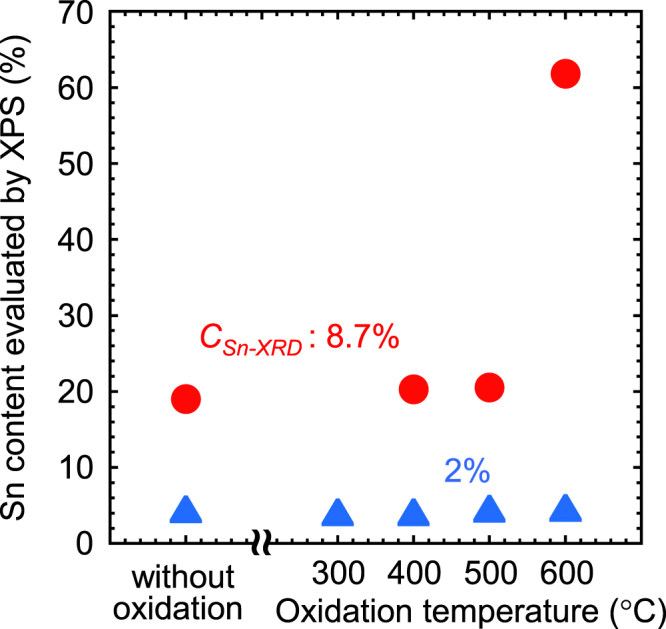
Sn contents (*C*_Sn-XPS_) evaluated from XPS as a function of the oxidation temperature.

A Ge_1−*x*_Sn_*x*_ layer with Sn contents of 2% or 8.7% formed on a Ge (001) substrate was thermally oxidized in dry oxygen ambient conditions at atmospheric pressure over a temperature range from 300 to 600 °C. Here, the thickness was set to 30 nm, which is much smaller than the thickness corresponding to the critical strain energy [102], to clarify the oxidation effect. The Ge_1−*x*_Sn_*x*_ layers were formed by MBE at 150 °C, and the Sn contents (*C*_Sn-XRD_) were estimated from XRD. Figure 8 shows XRD *ω*-2*θ* profiles measured near the 004 diffraction plane for the as-grown Ge_1−*x*_Sn_*x*_ layer and the Ge_1−*x*_Sn_*x*_ layers oxidized at 400 and 600 °C [21]. Here, *C*_Sn-XRD_ is 8.7%. A clear peak associated with 004 Ge_1−*x*_Sn_*x*_ diffraction in the *ω*-2*θ* profile for the as-grown Ge_1−*x*_Sn_*x*_ layer was observed at a diffraction angle of 32.2°. It should be noted that the oxidation at 400 °C only slightly induces a peak shift, although the peak width is broader than that for the as-grown layer; oxidation at 600 °C induces a slight peak shift toward the higher diffraction angle, which means precipitation of the substitutional Sn and/or strain relaxation by introducing misfit dislocations at the oxidation at 600 °C. Figure 9 shows the Sn content (*C*_Sn-XPS_) evaluated from x-ray photoemission spectroscopy (XPS) as a function of the oxidation temperature. The *C*_Sn-XPS_ values for the samples without oxidation are almost twice as large as the *C*_Sn-XRD_ values. This means that the surface Sn contents are higher than those inside the layers, which could be a result of the surface segregation during the Ge_1−*x*_Sn_*x*_ growth. For both samples, i.e., *C*_Sn-XRD_ of 2% and 8.7%, the Sn contents only change slightly after oxidation at less than 500 °C. However, after oxidation at 600 °C, the Sn contents increase, especially for a *C*_Sn-XRD_ of 8.7%, meaning that significant Sn segregation occurs. Taking into account the increase in *C*_Sn-XPS_ after oxidation at 600 °C, the peak shift shown in figure 8 could be a result of Sn precipitation, rather than the introduction of misfit dislocations.

**Figures 10.**
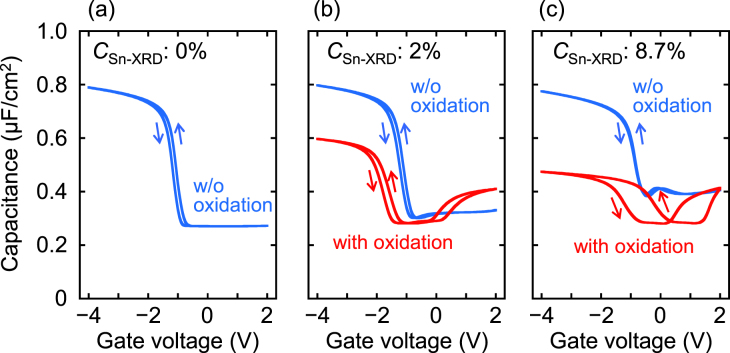
*C–V* curves for capacitors with *C*_Sn-XRD_ of (a) 0%, (b) 2% and (c) 8.7% with and without oxidation at 400 °C. Here, the measurement temperature and frequency were 100 K and 100 kHz, respectively.

The Sn segregation could have a significant impact on the electrical properties. Therefore, the *C*–*V* characteristics of Al/Al_2_O_3_/Ge_1−*x*_Sn_*x*_/Ge MOS capacitors with and without oxidized surfaces were investigated. Here, the Al_2_O_3_ layer was formed at a low temperature of 150 °C by atomic layer deposition to reduce Sn segregation during the Al_2_O_3_ layer formation. Figures 10(a)–(c) show *C–V* curves for capacitors with *C*_Sn-XRD_ of 0, 2, and 8.7%, respectively, with and without oxidation at 400 °C. Here, the measurement temperature and frequency were 100 K and 100 kHz, respectively. Additionally, *C*_Sn-XRD_ of 0% means homoepitaxial growth of Ge on a Ge substrate at 150 °C. The *C*–*V* curves without oxidation for all Sn contents indicate good MOS properties, with small hysteresis windows. Conversely, the *C*–*V* curves with oxidation have large hysteresis windows, especially in the case of a *C*_Sn-XRD_ of 8.7%. This means that slight changes in the Sn contents shown in figure 9 have large effects on the electrical interface properties. Therefore, suppression of the Sn segregation is very important for forming an insulator/Ge_1−*x*_Sn_*x*_ interface with high quality.

**Figures 11.**
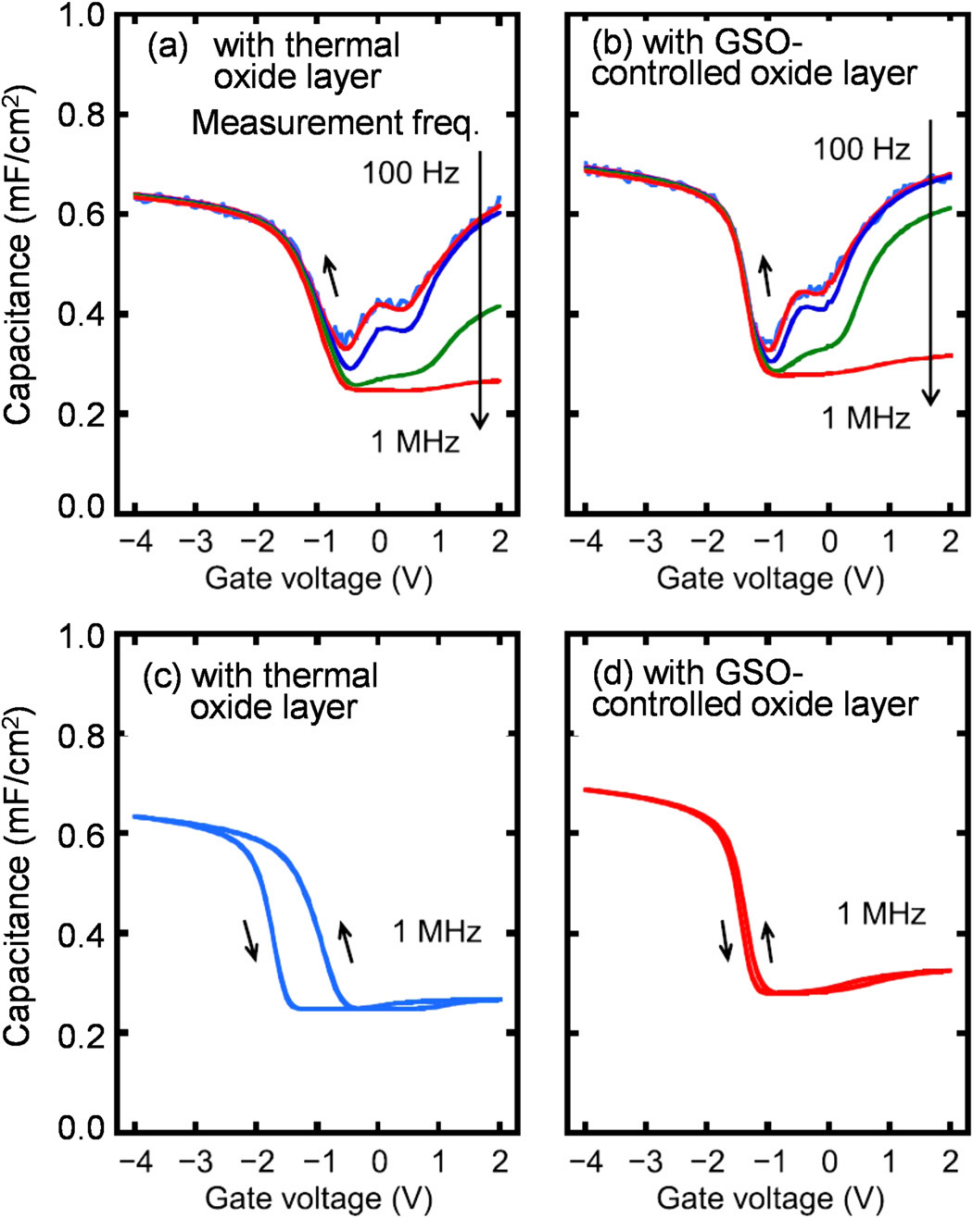
Comparison of the *C–V* characteristics of Al/Al_2_O_3_/Ge_0.924_Sn_0.076_/Ge MOS capacitors with (a) a thermal Ge_1−*x*_Sn_*x*_-oxide layer formed at 400 °C and (b) a GSO-controlled layer between the Al_2_O_3_ and Ge_0.924_Sn_0.076_ layers. 1 MHz *C–V* characteristics of the Al/Al_2_O_3_/Ge_0.924_Sn_0.076_/Ge MOS capacitors with (c) a thermal Ge_1−*x*_Sn_*x*_ oxide layer and (d) a GSO-controlled layer. Here, all *C*–*V* curves were measured at 100 K. Reprinted with permission from [95]. Copyright 2014, American Institute of Physics.

Although formation of a thin Ge-oxide layer between an insulator and Ge surface is very important for improving the MOS interface qualities, it is quite difficult to precisely control the thickness and to form the layer with high MOS interface quality because of thermal and chemical instabilities of the Ge-oxide layer formed on Ge [103, 104]. To overcome these problems, we have established a CVD method for formation of a thin Ge-oxide layer on a Ge substrate, which allows for controlling thickness on the atomic scale and suppression of Ge surface oxidation (GSO) because of the self-limiting adsorption of tetraethoxy-germanium on a Ge substrate and exposure of H_2_O, similar to the atomic layer deposition method [95]. Figures 11(a) and (b) compare the *C*–*V* characteristics of the Al/Al_2_O_3_/Ge_0.924_Sn_0.076_/Ge MOS capacitors with a thermal Ge_1−*x*_Sn_*x*_-oxide layer formed at 400 °C and a GSO-controlled layer, respectively, between the Al_2_O_3_ and Ge_0.924_Sn_0.076_ layers. Here, the Sn content was estimated by XRD. To match the maximum process temperature with the thermal oxidation, we performed the GSO-controlled deposition after N_2_ annealing at 400 °C for 10 min. Near a gate voltage (*V*_*g*_) = −1 V, the frequency dispersion for the thermally oxidized sample was larger than that for the GSO-controlled sample. In fact, the *D*_it_ values of the thermally oxidized and GSO-controlled deposition samples, evaluated by the conductance method at *C*/*C*_ox_ ∼ 0.45, were 1.2 × 10^12^ and 7.2 × 10^11^ cm^−2^eV^−1^, respectively. Here, *C* and *C*_ox_ mean the measured capacitance and oxide capacitance, respectively. These results indicate that the GSO-controlled deposition decreases *D*_it_. Figures 11(c) and (d) show the 1 MHz *C–V* characteristics of Al/Al_2_O_3_/Ge_0.924_Sn_0.076_/Ge MOS capacitors with a thermal Ge_1−*x*_Sn_*x*_ oxide layer and a GSO-controlled layer, respectively. The measurement temperature was 100 K. Although these samples did not differ much in *D*_it_, the hysteresis width of the sample with the GSO-controlled layer was much smaller than that of the thermally oxidized sample. Considering that hysteresis originates from slow states formed at the Al_2_O_3_/Ge-oxide interfaces, this result indicates that forming the oxide layer by GSO-controlled deposition effectively reduced the slow state density.

**Figure 12.**
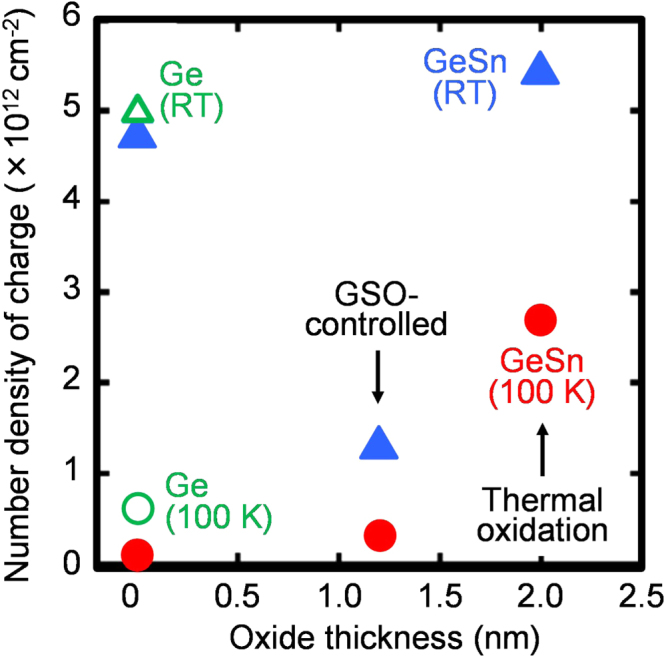
The charge density evaluated from the hysteresis width at *C*/*C*_ox_ = 0.75 and the *C*_ox_ value in the *C–V* characteristics as a function of the Ge or Sn oxide thicknesses. Here, the thicknesses of the oxide layers of 0, 1.2 and 2.0 nm correspond to the samples after HF treatment, with the GSO-controlled layer and with the thermal oxide layer, respectively. Reprinted with permission from [95]. Copyright 2014, American Institute of Physics.

Figure 12 shows the number density of charges evaluated from the hysteresis width at *C*/*C*_ox_ = 0.75 and the *C*_ox_ value in the *C–V* characteristics as a function of the Ge or Sn oxide thickness. Here, we evaluated the oxide thicknesses of the oxide/Ge_1−*x*_Sn_*x*_ samples without an Al_2_O_3_ layer by XPS. At an oxide thickness of ∼0 nm, the charge densities for the Al_2_O_3_/Ge_0.924_Sn_0.076_/Ge stack were almost identical to those for the Al_2_O_3_/epitaxial-Ge/Ge stack. This implies that the MOS slow-states were not significantly affected by the existence of Sn atoms. In addition, the charge densities as a function of the oxide thickness do not show a linear relationship between the oxide thickness and the charge density, implying that the thermal oxidation enhances Sn migration, which leads to complicated defect structures, inducing slow states. Furthermore, both charge densities at RT and 100 K for the GSO-controlled deposition are smaller than those for thermal oxidation. This could occur because atomically controlled deposition by the GSO-controlled deposition suppressed the Sn migration. Consequently, suppressing Sn migration is important for forming Ge_1−*x*_Sn_*x*_ gate stacks with low densities of slow states and interface states.

### 4.3. Formation and properties of metal/Ge_1−*x*_Sn_*x*_ contacts

The formation of metal/Ge_1−*x*_Sn_*x*_ contacts is one of the critical issues in the realization of high-performance electronic devices. For metal/semiconductor contacts, there are some requirements: (1) low parasitic resistance, including low contact resistivity of the metal/semiconductor interface; (2) small resistivity of the metal electrode; (3) shallow contact structure with homogeneous and flat interface; (4) high thermal robustness for post-thermal processes; and (5) high uniformity of not only the crystalline structure but also the electronic properties. To lower the contact resistivity of a metal/semiconductor contact, it is necessary to lower the Schottky barrier height (SBH) at the interface and to increase the carrier density by increasing the doping concentration in the semiconductor. The SBH of metal/Ge_1−*x*_Sn_*x*_ interfaces has not been clarified in detail yet, and the doping technology for Ge_1−*x*_Sn_*x*_ should be developed.

Metal germanide, which is reaction product of a metal and Ge, is generally used for contact materials for device applications. NiGe is a promising candidate for metal germanide materials because it has some advantages compared with various metal germanides, including low resistivity, low formation temperature (below 400 °C), low Ge consumption in the formation of monogermanide, and the thermal stability of the final phase in Ni–Ge binary systems [105, 106].

Previously, we investigated the thin film formation of the reaction product, Ni germanostanane, with a solid-phase reaction in an Ni/Ge_1−*x*_Sn_*x*_ contact with various Sn contents [107]. The formation of poly-crystalline Ni(Ge_1−*x*_Sn_*x*_) thin films is observed after the annealing of Ni/Ge_1−*x*_Sn_*x*_/Ge(001) samples with Sn contents ranging from 2.0 to 6.5% at a temperature ranging from 350 to 550 °C, like the NiGe formation in a Ni/Ge system. The lattice parameters of the Ni(Ge_1−*x*_Sn_*x*_) layer show anisotropic deformation because of the incorporation of Sn into NiGe, especially after low-temperature annealing at 350 °C. The lattice parameter approaches that of NiGe after annealing at a higher temperature (650 °C), suggesting Sn precipitation from the substitutional site of Ni(Ge_1−*x*_Sn_*x*_). We also demonstrate that the uniform and flat morphology of the Ni(Ge_1−*x*_Sn_*x*_) layer can be achieved by annealing the Ni/Ge_1−*x*_Sn_*x*_/Ge(001) at a low temperature of 350 °C, even with a high Sn content of 6.5%. However, agglomeration of the poly-Ni(Ge_1−*x*_Sn_*x*_) film is an issue [107, 108], like poly-NiGe films on Ge substrates [106]. The agglomeration of poly-Ni(Ge_1−*x*_Sn_*x*_) causes an increase in the sheet resistance of the germanostanane film [109]. In particular, the agglomeration of Ni(Ge_1−*x*_Sn_*x*_) is enhanced with increasing Sn content, which can be attributed to the low melting temperature of Sn and the low eutectic temperatures of Sn–Ge and Sn–Ni systems.

There have been some proposals for the improvement of the thermal stability of poly-Ni(Ge_1−*x*_Sn_*x*_) films on Ge_1−*x*_Sn_*x*_ by incorporating an additional element. The incorporation of Pt into Ni(Ge_1−*x*_Sn_*x*_) can effectively enhance the thermal stability [109], similar to the poly-NiGe film on Ge [110, 111]. The incorporation of Pt with a ratio of 1/3 to the Ni layer on the Ge_0.947_Sn_0.053_ epitaxial layer and annealing at 350–550 °C causes the formation of a polycrystalline film that consists of Ni(GeSn) + Pt_*x*_(GeSn)_*y*_, and the thermal stability of the polycrystalline film is improved up to 500 °C. C pre-implantation to Ge_1−*x*_Sn_*x*_ also effectively improves the thermal stability of the poly-Ni(Ge_1−*x*_Sn_*x*_) layer on Ge_1−*x*_Sn_*x*_ [112]. It is known that C incorporation into Ge substrates improves the thermal stability of poly-NiGe films on Ge substrate [113, 114], and this technology should also be available in poly-Ni(Ge_1−*x*_Sn_*x*_)/Ge_1−*x*_Sn_*x*_ systems.

The existence of Sn significantly influences the crystal growth and reactions, including germanidation, and also affects the electrical properties of metal/Ge contacts. Koike *et al* reported that germanidation with annealing at 350 °C of a Ni/Sn bilayer on a Ge(001) substrate causes the epitaxial growth of a NiGe layer [115]. This epitaxial NiGe layer has a uniform NiGe/Ge(001) interface with atomic flatness. In addition, this epitaxial NiGe/n-Ge(001) contact shows an SBH reduction of 0.1 eV compared with a conventional NiGe/n-Ge(001) contact.

The control of the SBH at metal/Ge interfaces is one of the most important issues in reducing the contact resistivity for realising high-performance Ge nanoelectronic devices. However, it is difficult to control the SBH of metal/n-Ge contacts because the Fermi level pinning (FLP) phenomenon takes place at metal/Ge interfaces. The Fermi level of metals on Ge is generally pinned near the valence band edge of Ge, and the SBH of metal/n-Ge usually has a value higher than 0.5 eV [116, 117]. There are few reports of the SBH at metal/Ge_1−*x*_Sn_*x*_ interfaces, but it is important for understanding the electrical properties of the interface.

Recently, we found a smaller SBH in Sn/n-Ge contacts prepared by Sn deposition at RT compared with conventional metal/n-Ge contacts in which the FLP occurs [118]. The SBH of the Sn/n-Ge interface was estimated to be as low as 0.35 eV from the current–voltage characteristics of the Sn/n-Ge(001) Schottky diode. The hard x-ray photoelectron spectroscopy measurement of the energy band bending of Ge near the Sn/n-Ge interface supports this reduction of SBH. The theoretical calculations from Nakayama’s group predict that the FLP can be alleviated by the formation of *α*-Sn at the *β*-Sn/Ge interface [119]. A comprehensive study of the crystalline and electrical properties of Sn/Ge and metal/Ge_1−*x*_Sn_*x*_ contacts is continuously required for device applications of Ge_1−*x*_Sn_*x*_-related materials.

### 4.4. Defect properties of Ge_1−x_Sn_x_

In an epitaxial Ge or Ge_1−*x*_Sn_*x*_ layer, holes are unintentionally generated because of defects with shallow energy levels close to the valence band edge [16, 120]. Although the physical origin of such generated holes remains unclear, one possibility for the Ge layer is multi-vacancy complexes, which form acceptor-like states with energy levels of *E*_*v*_ + (10 – 20) meV [121]. Here, *E*_*v*_ is the energy level of the valence band edge of Ge.

Defects can be also induced by plasma processes, doping, growth, and the annealing process during device fabrication in Ge and Ge_1−*x*_Sn_*x*_ layers. The induced defects and unintentionally generated holes can degrade device performance; for example, there is a concerning increase in the dark current in Ge_1−*x*_Sn_*x*_ photodetectors and junction leakage currents in Ge_1−*x*_Sn_*x*_ MOSFETs and TFETs. Therefore, control of the defect structure, defect concentration, and energy level of defect states in Ge and Ge_1−*x*_Sn_*x*_ layers and the Ge substrate is important for realising Ge and Ge_1−*x*_Sn_*x*_ devices. Recently, we investigated the impact of the plasma process on the defect density, defect structures, and energy levels in Ge compared with Si and the interaction of Sn and hydrogen with defects. This section consists of four sub-sections including our recent results: (1) the origin and activation energy of defects derived from Ge–Ge bonds in Ge; (2) the electrical properties of undoped Ge_1−*x*_Sn_*x*_ epitaxial layers; (3) a theoretical calculation of defects in Ge_1−*x*_Sn_*x*_; and (4) observations of Sn-related defects in Ge and how to recover the crystallinity with annihilating defects.

**Figure 13.**
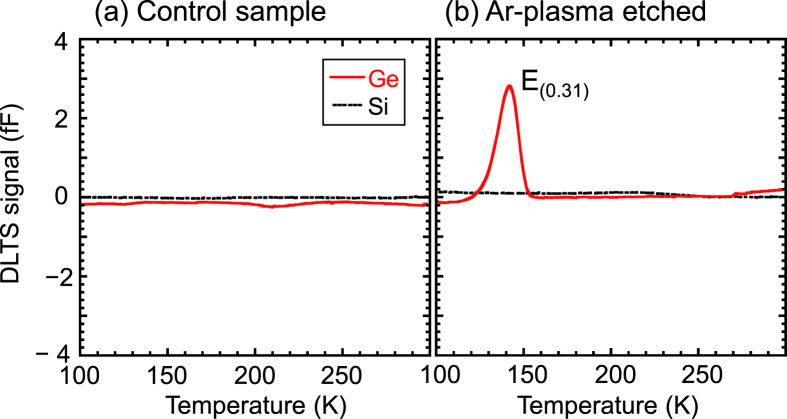
DLTS spectra for Ge and Si Schottky diodes: (a) control sample; (b) Ar-plasma-etched samples.

First, we discuss the origin and activation energy of defects derived from Ge–Ge bonds in Ge. The Ge–Ge bond energy (1.61 eV) is smaller than the 1.8 eV for Si–Si bonds. [122]. Because these bonds are weaker than those of Si, they necessitate elaborate control over defect formation. In Ar plasma etching, defects whose energy states of *E*_*c*_ − 0.31 eV were in the forbidden band of Ge were induced during etching, while they were not detected in Si with the same conditions, as shown in figure 13 [123]. Furthermore, similar defects are induced with exposure of Ge to He or H_2_ plasma [124]. Various defects in Ge are created during not only the plasma etching but also the electron or alpha-ray irradiation and the electron beam deposition of metal [125]. The origins of these defects may be vacancies, interstitials, impurities, or various vacancy complexes, among others. Table 1 shows a summary of the defects observed using the deep level transient spectroscopy (DLTS) technique [124–136]. Thus, elaborate control of the defect formation in the Ge LSI process is necessary because Ge–Ge bonds are weaker than those in the conventional Si case.

**Table 1.** Activation energy of various process induced defects in Ge observed by DLTS.

Defects		Activation energy (eV)		Sample condition
		Electron	Hole	
	I related	0.04		p-irradiation^l^
I related	I_Ge_ (self-interstitial)	0.11		e-irradiation^a^
	V(0/−)		0.02	Quenching^n^
	V(−/2−)		0.26	Quenching^n^
Vacancy related	V_2_	0.29^g^^,^^m^, 0.31^j^		p-^m^, e-^g^, n^g^- irradiation, SD^j^
	V_2_(2−/0)		0.19	2MeV p- or e- irradiation^i^
	Small vacancy cluster	0.1		n- irraduated^g^
	Sb-V(+/0)		0.09	e-irradiation^l^,EBD^j^
	Sb-V(0/−)		0.30^j^, 0.307^d^, 0.31^j^	a^j^-, e^j^- irradiation, EBD^j^
	Sb-V(2−/−)	0.37^m^, 0.377^d^, 0.38^g^^,^^j^^,^^l^		e^g^^,^^j^^,^^l^-, n^g^-, p^l^- irradiation, EBD^j^
		0.13^j^^,^^m^, 0.14^j^, 0.15^l^		P^l^^,^^m^-, e^l^^,^^m^- irradiation, SD^j^
Sb related	Sb and I related	0.19^m^, 0.20^j^^,^^l^, 0.23^m^^,^^l^		a^j^-,p^l^^,^^m^-, e^j^^,^^l^^,^^m^- irradiation, SD^j^
	Sb related	0.21^j^^,^^m^, 0.31^k^	0.30^m^	a^j^-, p^m^-, e^j^^,^^m^- irradiation, Ar ICP etching^k^, H or He-plasma^k^
	V-Sb_2_		0.27	e- or p-irradiation^l^
	P-V(−/0)		0.35	e- irradiation^f^
P related	V-P(2−/−)	0.293		e- irradiation^f^
	VO(2−/−)	0.21^c^, 0.27^m^, 0.285^a^		*γ*^c^, p^m^-, e^a^^,^^m^- irradiation
	VO(−/0)		0.27	*γ*-irradiation^c^
	VO_2_(2−/−)	0.195		e-irradiated oxygen-rich Ge^b^
O related	VO_2_(0/−)	0.365		e-irradiated oxygen-rich Ge^b^
	O related	0.14, 0.19		p-, e-irradiation^m^
	O, H related		0.15	p-irradiation^l^
	I_Ge_–O_2i_	0.062, 0.080		4 MeV e-irradiatied oxygen-rich Ge:Sb^a^
	Sn-V(2−/−)		0.19	e-irradiated Ge:Sn + P^f^
	SnVP complex		0.21	e-irradiated Ge:Sn + P^f^
Sn related	Sn related (GeSn:P)	0.11, 0.27		e-irradiated Ge:Sn + P^f^
	SnPV		0.208	e-irradiated Ge:Sn + P^h^

Reference numbers corresponding to index are ^a^ [126], ^b^ [127], ^c^ [128], ^d^ [129], ^e^ [128], ^f^ [130], ^g^ [131], ^h^ [132], ^i^ [133], ^j^ [125], ^k^ [124], ^l^[134], ^m^ [135], and ^n^ [136].

a: alpha, p: proton, e: electron, *γ*: gamma-ray, n: neutron, SD: sputter deposition, EBD: electron beam deposition.

**Figure 14.**
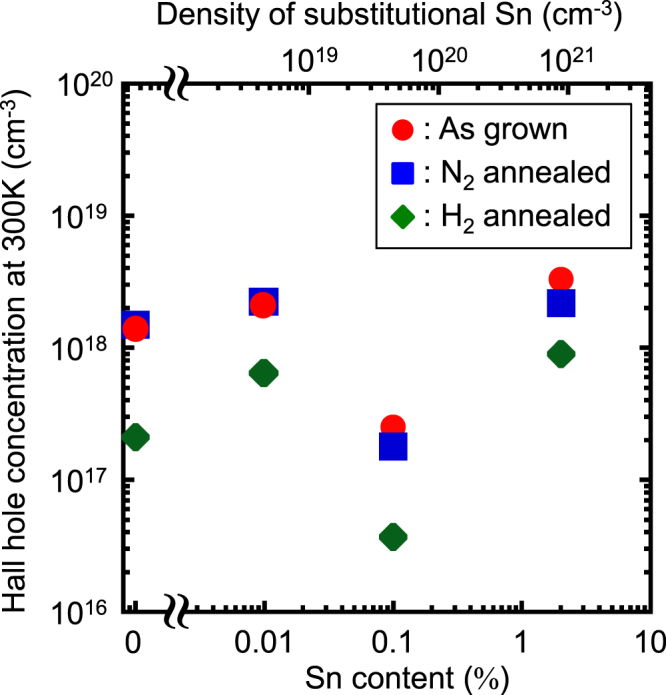
Sn content dependence of Hall hole concentration in undoped Ge_1−*x*_Sn_*x*_ layers on SOI substrates. Reprinted from [16], Copyright 2013, with permission from Elsevier.

Second, we discuss the electrical properties of undoped Ge_1−*x*_Sn_*x*_ epitaxial layers. The influence of Sn on defect formation in Ge_1−*x*_Sn_*x*_ alloy thin films is a concern. *α*-Sn is a group-IV semiconductor material similar to Ge, and a substitutional Sn atom in a Ge matrix is an electrically neutral isovalent impurity. Low-temperature growth is an important factor required for Ge_1−*x*_Sn_*x*_ growth to realize a high Sn content, and point defects, such as atomic vacancies, are easily induced in the growth layer because the diffusion and reconstruction of Ge and Sn atoms rarely occur during the low-temperature growth. Sn atoms with a large covalent radius are considered to be stabilised by the formation of Sn–V pairs in a Ge matrix [28]. These vacancy defects should influence the electrical properties of Ge_1−*x*_Sn_*x*_ layers. In fact, it is reported that an undoped Ge_1−*x*_Sn_*x*_ epitaxial layer shows p-type conduction with a high concentration of unintentionally generated holes, as shown in figure 14 [16]. The high hole concentration only changes slightly, even after annealing at 500 °C in a N_2_ atmosphere. In contrast, after H_2_ annealing at 500 °C, the hole concentration decreases by approximately one order of magnitude compared to that with N_2_ annealing. The minimum value of the carrier concentration is 3.7 × 10^16^ cm^−3^ at an Sn content of 0.1%. Additionally, shallow and deep level defects are found in an unintentionally doped Ge_1−*x*_Sn_*x*_ (*x* = 0.06%) layer grown on n^-^-Si substrate by UHVCVD, and their activation energies were estimated to be 7.5 and 140 meV, respectively [120]. However, the origin of the carrier generation and the properties of defects related to Sn in Ge_1−*x*_Sn_*x*_ have not yet been understood in detail.

Next, we introduce recent theoretical calculations of defects in Ge_1−*x*_Sn_*x*_. Sn impurity atoms are known to be very effective traps for vacancies and impurities. It is thought that an Sn atom in Ge traps vacancies by forming an Sn–V pair, and the binding energies of the Sn–V and V–V complexes are theoretically estimated to be 0.61 and 0.48 eV, respectively (see table 2 [137, 138]), meaning that the Sn–V complex is more stable than the V–V complex [30]. The theoretical calculations predict that when single Sn–V pairs or Sn–V complex pairs are formed in Ge and Si, Sn atoms become stable in terms of the energy. Oversize impurities such as Sn in Ge are located between two semivacancies, i.e*.*, the split-V configuration [139]. Additionally, theoretical results predict the formation of Sn_n_V_m_ clusters that are more stable than V–V (see table 2). With high Sn contents in Ge, the Sn_n_V_m_ clusters are likely to form [137]. Furthermore, it has been reported that the calculated binding energies of SnVO and A centers (VO) are 1.09 and 0.45 eV, respectively. This means that the SnVO center is more stable than the A center [140].

**Table 2.** Predicted binding energies, *E*_b_ of defect complex and Sn_n_ V_m_ complexes in Ge.

Defect complex	*E*_b_ (eV)
VV	−0.48^a^
SnV	−0.61^a^ (−0.64^b^)
PV	−0.52^b^
AsV	−0.60^b^
SbV	−0.70^b^
SnSn	0.03^a^
SnVV	−1.10^a^
VSnV	−0.79^a^
VSnSn	−1.00^a^
SnVSn	−0.97^a^

Reference numbers corresponding to index are ^a^ [137] and ^b^ [138].

**Figure 15.**
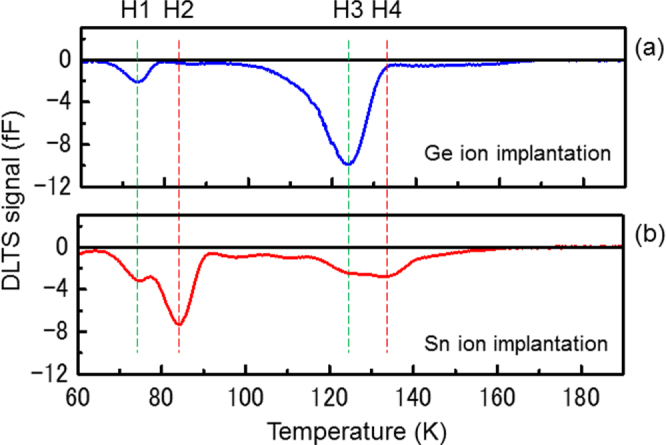
DLTS spectra of Ge samples implanted with (a) Ge and (b) Sn to a dose of 1 × 10^14^ cm^−2^ after N_2_-annealing.

Here, we introduce some observations of Sn-related defects in Ge and discuss how to recover the defects. To control defects in Ge_1−*x*_Sn_*x*_, it is necessary to identify the defects and interactions of defects with vacancy complexes, the matrix, and impurities. Recently, a defect level of Sn–V was experimentally estimated to be as shallow as *E*_*v*_ + 0.19 eV in electron-irradiated Ge with doping by Sn and phosphorus (P) using DLTS [130]. Additionally, a defect of SnV_2_^0^Ga has been reported in electron-irradiated Ga and Sn-doped Ge using FT-IR. [141]. The Sn and vacancy complex formed not only Sn–V but also Sn_n_V_m_ clusters. After annealing at temperatures higher than 125 °C, an SnVP complex is formed by the interaction of mobile V–P complexes with Sn [132]. Furthermore, Sn–V-related defects have been observed in an Sn ion-implanted Ge, as shown in figure 15 [142]. Sn ion implantation with a dose of 1 × 10^14^ cm^−2^ into n-Ge(Sb doped) reduces the concentration of the Sb–V defect (H4), while a new defect (H5) is formed.

In addition, the doping of Ge with Sn leads to a change in the reaction involving oxygen and vacancies. FT-IR measurements reveal that Sn atoms interact with oxygen–vacancy (V–O) centers (the peak positions are 621 and 669 cm^−1^), forming an SnVO center (the peak positions are 501.6 and 604.2 cm^−1^) [143]. The SnVO center in Ge is more stable than the V–O center, which is formed with annealing at a temperature range from 360 to 493 K. Based on these results, it is clear that Sn atoms preferentially interact with vacancies, vacancy–dopant (V–P, V–Sb) complexes, and V–O centers.

**Figures 16.**
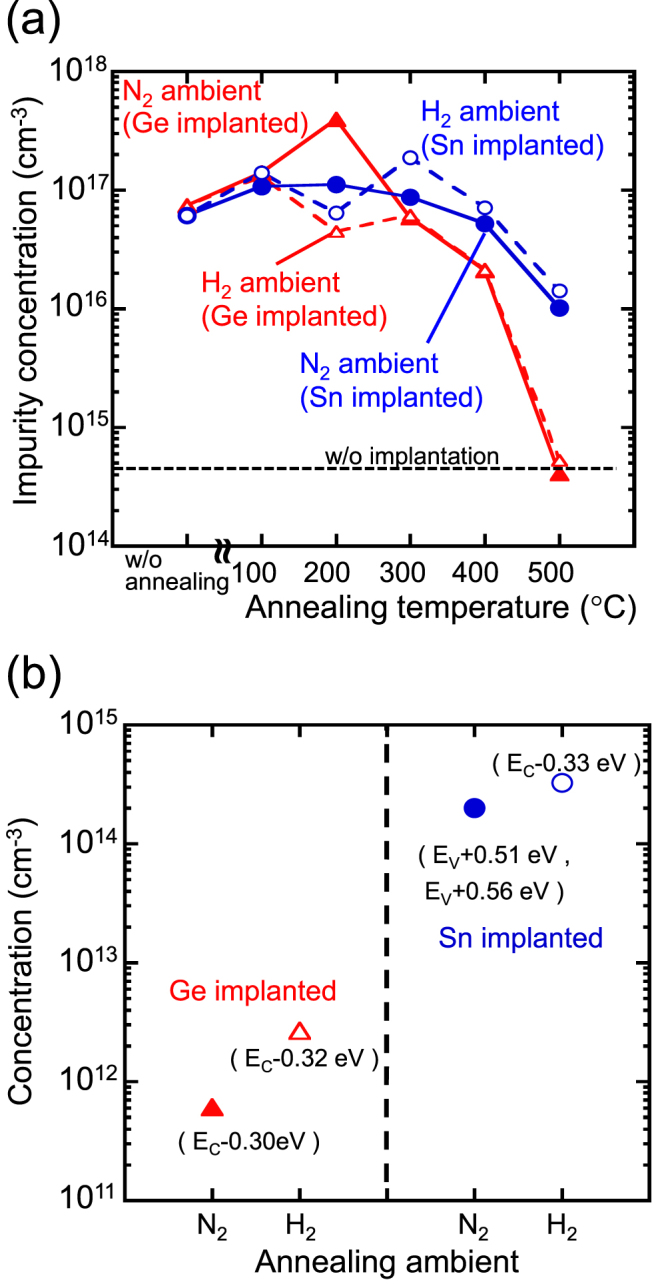
(a) Annealing temperature dependence of the impurity concentrations evaluated from the 1/*C*^2^–*V* characteristics. (b) Defect concentrations of the Sn- and Ge-implanted samples annealed at 500 °C in N_2_ or H_2_ ambient condition.

To recover the crystallinity of Ge by annihilating defects, it is important to choose a suitable annealing temperature and annealing atmosphere. Figure 16(a) shows the annealing temperature dependence of the impurity concentration evaluated from the slopes of the 1/*C*^2^–*V* characteristics for Sn- and Ge-implanted n-Ge(Sb doped) substrate annealed in N_2_ or H_2_ [144]. It should also be noted that for the annealing temperature at 500 °C, the impurity concentration in the Ge-implanted samples is identical to that for the non-implanted one, while the concentration in the Sn-implanted samples is still higher than that for the non-implanted one. In DLTS measurements of these samples, defects with different structures were observed in the Sn-implanted samples, as shown in figure 16(b). The energy levels of the defects were *E*_*c*_ − 0.33 eV from the conduction band edge and *E*_*v*_ + 0.56 eV from the valence band edge. This strongly suggests that defects induced by ion implantation can interact with Sn atoms during high-temperature annealing, and the defect concentration shows a slight decrease, even after annealing below 500 °C. These results suggest the presence of defects that interact with Sn or hydrogen atoms, which indicates the possibility of defect control in Ge substrates by Sn or hydrogen incorporation. This defect control could yield high-performance Ge_1−*x*_Sn_*x*_-based devices.

## 5. Device applications of Ge_1−*x*_Sn_*x*_-related materials

### 5.1. Electronic device applications

**Figure 17.**
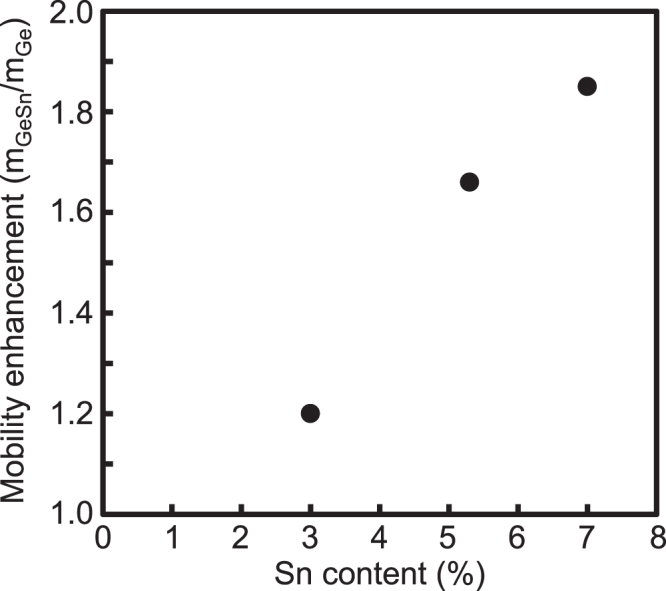
Mobility enhancement, which was determined by the *μ*_GeSn_ of a Ge_1−*x*_Sn_*x*_ MOSFET divided by the *μ*_Ge_ of a Ge MOSFET, as a function of Sn content in a channel.

Control of the energy band structures and the effective mass for Sn-related alloys by Sn incorporation into Ge and Si_1−*x*_Ge_*x*_ makes it possible to realize not only high-performance MOSFETs but also low-power consumption devices. The operation of n-channel and p-channel Ge_1−*x*_Sn_*x*_ MOSFETs has been reported [145, 146]. However, the performances are not as high as those expected from the Ge_1−*x*_Sn_*x*_ material properties [147, 148]. Although clear physical origins of these inadequate performances have not been clarified yet, the inadequate quality of the gate stack structures attributed to the Sn migration, as discussed in section 4, could be one of the reasons for this performance. However, comparative studies of the effective mobilities of Ge and Ge_1−*x*_Sn_*x*_ MOSFETs with the same fabrication process revealed a possibility for realising high-performance p-channel Ge_1−*x*_Sn_*x*_ MOSFETs. Figure 17 shows the mobility enhancement, which was determined by the effective hole mobility (*μ*_GeSn_) of a Ge_1−*x*_Sn_*x*_ MOSFET divided by the effective hole mobility (*μ*_Ge_) of a Ge MOSFET, as a function of Sn content in the channel. The mobility enhancement increases with the Sn content, indicating the effectiveness of an Sn alloy for high-performance MOSFETs. We hope that, in the near future, an optimized fabrication process and new technologies will lead to further improvement of the device performance.

**Figure 18.**
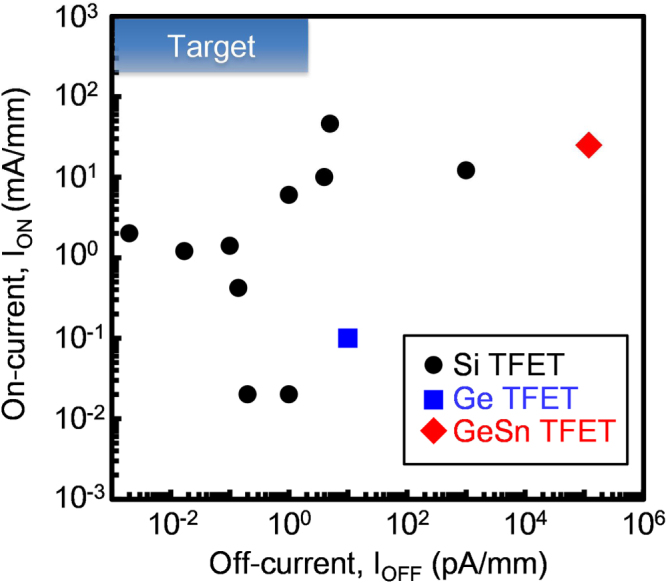
Relationships between *I*_ON_ and *I*_OFF_ for various TFETs with Si, Ge and Ge_1−*x*_Sn_*x*_ channels.

Because conventional MOSFET operation is limited by carrier diffusion near the subthreshold region, which means that the minimum switching voltage of a conventional MOSFET is determined by its operation temperature, a new physical principle for device operation is required for low-power consumption systems operating at voltages less than 0.2–0.3 V. A TFET, which uses band-to-band tunneling to obtain a drive current, is a candidate for switching devices in such a system. The drive current strongly depends on the tunneling probability, i.e., the magnitude of the bandgap. Although a small bandgap is an advantage for obtaining a high on-current (*I*_ON_), the small bandgap also induces a high off-current (*I*_OFF_). Recently, many demonstrations of TFET operations for various device structures and various materials have been reported. Figure 18 shows relationships between *I*_ON_ and *I*_OFF_ for various TFETs with Si, Ge, and Ge_1−*x*_Sn_*x*_ channels [149–160]. The blue square is the target region for switching devices in a low-power consumption system. Here, the upper limit of *I*_OFF_ is determined by the *I*_OFF_ for low-power devices provided in the international technology roadmap for semiconductors 2013 [161]. The lower limit of the *I*_ON_ could be determined by the application. It should be noted that present TFETs have not achieved the target. Furthermore, Ge_1−*x*_Sn_*x*_ TFETs have a quite large *I*_OFF_, which can be attributed to the small bandgap of Ge_1−*x*_Sn_*x*_ and defects in the epitaxial Ge_1−*x*_Sn_*x*_ layer. To realize TFETs, further improvements in the device structure and control of the energy band structures in Sn-related alloys with high crystalline quality are needed.

### 5.2. Optoelectronic device applications

Various optoelectronic applications of Ge_1−*x*_Sn_*x*_, such as photodiodes, LEDs, lasers, and photovoltaic cells, have been reported. In these applications, the features of Ge_1−*x*_Sn_*x*_ are used for energy band engineering [162–167]. Energy band narrowing with Ge_1−*x*_Sn_*x*_ extends the available wavelength to a longer range compared with Ge photoelectronic devices. In addition, the indirect to direct cross over in Ge_1−*x*_Sn_*x*_ with a high Sn content up to approximately 8% significantly improves the photoresponse and photoabsorbance. Additionally, Ge_1−*x*−*y*_Si_*x*_Sn_*y*_ ternary alloys provide much more capability for energy band design and lattice control technology similar to III–V compound semiconductors.

Ge_1−*x*_Sn_*x*_ photodetectors provide an improved photoresponse at longer IR wavelengths with increasing Sn content [162–164]. A photodetector with a Ge_1−*x*_Sn_*x*_/Ge QW structure on an SOI substrate has been also reported [165]. A Ge_1−*x*_Sn_*x*_ heterojunction LED has been demonstrated with Ge_1−*x*_Sn_*x*_ epitaxial layers on an Si substrate prepared using low-temperature MBE [166]. The Ge_1−*x*_Sn_*x*_ LED shows a direct bandgap electroluminescence at RT, increasing in intensity with increasing Sn content.

Theoretical calculations for laser applications of Ge_1−*x*_Sn_*x*_ and Ge_1−*x*−*y*_Si_*x*_Sn_*y*_ have been reported [85, 168–172]. Ge_1−*x*_Sn_*x*_/Ge_1−*x*−*y*_Si_*x*_Sn_*y*_ heterojunction QW structures are designed for the active region, and the energy band structure, carrier lifetime, optical confinement, and modal gain have been theoretically calculated. Recently, a practical experimental study of lasing in direct-bandgap Ge_1−*x*_Sn_*x*_ on a Ge virtual substrate was conducted [167]. The laser action has been observed under optical pumping in a patterned Ge_1−*x*_Sn_*x*_ cavity at low temperatures, below 90 K.

Energy band engineering with ternary alloys of group-IV materials is promising for solar cell applications. Kouvetakis’s group proposed the application of a Ge_1−*x*−*y*_Si_*x*_Sn_*y*_ buffer layer for multi-junction solar cells with III–V compound semiconductors [75]. A Ge_1−*x*−*y*_Si_*x*_Sn_*y*_ epitaxial layer whose lattice matches that of a Ge substrate with an energy bandgap of 1.0 eV is suitable for use as the buffer layer for an In_1−*x*_Ga_*x*_As layer. [173] Recently, we also proposed a multi-junction cell with all group-IV semiconductor alloys, including Ge_1−*x*−*y*_Si_*x*_Sn_*y*_, Ge_1−*x*−*y*_C_*x*_Sn_*y*_, and Si_1−*x*−*y*_Sn_*x*_C_*y*_ [81, 86–88]. We investigated the solar cell actions of Ge_1−*x*−*y*_Si_*x*_Sn_*y*_/Ge heterojunction structures and observed the enhancement of the external quantum efficiency at an energy of 1.0 eV, corresponding to the energy bandgap of the Ge_1−*x*−*y*_Si_*x*_Sn_*y*_ epitaxial layer [174, 175].

## 6. Conclusions

We have reviewed the crystalline and electronic properties of Ge_1−*x*_Sn_*x*_-related materials and introduced some recent technological achievements in crystal growth, interface engineering, and device applications. Ge_1−*x*_Sn_*x*_ provides novel engineering technology of the strain structure and energy band alignment for Si nanoelectronics. The direct–indirect crossover in Ge_1−*x*_Sn_*x*_ with a high Sn content is promising for new applications in IR optoelectronics consisting of only group-IV semiconductor materials. Sn incorporation into the crystal growth of Si, Ge, and Si_1−*x*_Ge_*x*_ improves not only the growth temperature, crystalline structure, and defect control, but also the electronic properties of Ge_1−*x*_Sn_*x*_-related thin films. The interface engineering of Ge_1−*x*_Sn_*x*_-related materials is a critical issue, and future investigations of the solid-phase reaction and the electronic properties of oxide/semiconductor and metal/semiconductor interfaces are required. Ternary alloy materials related to Ge_1−*x*_Sn_*x*_ are promising for extensive applications in Si electronics and optoelectronics, especially for realising type-I energy band alignment with unstrained Ge_1−*x*−*y*_Si_*x*_Sn_*y*_/Ge heterointerfaces, which should extend the various applications of Si-based nanoelectronics. The science and technology of Ge_1−*x*_Sn_*x*_-related materials are expected to be widely and increasingly developed in the future, providing further innovation for multifunctional, low-power, high-speed, and integrated electronic devices.
